# Complementary Patterns of Direct Amygdala and Hippocampal Projections to the Macaque Prefrontal Cortex

**DOI:** 10.1093/cercor/bhv019

**Published:** 2015-02-24

**Authors:** John P. Aggleton, Nicholas F. Wright, Douglas L. Rosene, Richard C. Saunders

**Affiliations:** 1 School of Psychology, Cardiff University, Cardiff, WalesCF10 3AT, UK; 2 School of Medicine, Department of Anatomy and Neurobiology, Boston University, Boston MA 02118, USA; 3 Laboratory of Neuropsychology, National Institute of Mental Health, Bethesda, MD 20892, USA

**Keywords:** anatomy, emotion, fornix, hippocampus, memory, subiculum

## Abstract

The projections from the amygdala and hippocampus (including subiculum and presubiculum) to prefrontal cortex were compared using anterograde tracers injected into macaque monkeys (*Macaca fascicularis*, *Macaca mulatta*). Almost all prefrontal areas were found to receive some amygdala inputs. These connections, which predominantly arose from the intermediate and magnocellular basal nucleus, were particularly dense in parts of the medial and orbital prefrontal cortex. Contralateral inputs were not, however, observed. The hippocampal projections to prefrontal areas were far more restricted, being confined to the ipsilateral medial and orbital prefrontal cortex (within areas 11, 13, 14, 24a, 32, and 25). These hippocampal projections principally arose from the subiculum, with the fornix providing the sole route. Thus, while the lateral prefrontal cortex essentially receives only amygdala inputs, the orbital prefrontal cortex receives both amygdala and hippocampal inputs, though these typically target different areas. Only in medial prefrontal cortex do direct inputs from both structures terminate in common sites. But, even when convergence occurs within an area, the projections predominantly terminate in different lamina (hippocampal inputs to layer III and amygdala inputs to layers I, II, and VI). The resulting segregation of prefrontal inputs could enable the parallel processing of different information types in prefrontal cortex.

## Introduction

The prefrontal cortex is assumed to orchestrate multiple classes of information to maintain cognitive control (Miller and Cohen 2001). Among its many afferents are direct projections from the amygdala and hippocampus. These medial temporal lobe connections have long been implicated in a wide array of affective and cognitive processes (e.g., Delamillieure et al. 2002; Simons and Spiers 2003; Van Elst et al. 2005; Bachevalier and Loveland 2006; Phelps 2006; Bishop 2007; Milad and Rauch 2012; Preston and Eichenbaum 2013; Rhodes and Murray 2013; Ruff and Fehr 2014). Consequently, the detailed topography of these prefrontal inputs remains of considerable interest.

It is already known in Old World monkeys that projections from the amygdala terminate widely across prefrontal cortex (Amaral and Price 1984; Barbas and Olmos 1990; Morecraft et al. 1992; Carmichael and Price 1995; Ghashgaei et al. 2007), whereas hippocampal projections appear more restricted, with inputs focused on medial (areas 25 and 32) and orbital (areas 11, 13, and 14) prefrontal cortex (Rosene and Van Hoesen 1977; Morecraft et al. 1992; Barbas and Blatt 1995; Carmichael and Price 1995; Insausti and Munoz 2001). Most of these connection studies have, however, relied on placing retrograde tracers within different parts of prefrontal cortex, with the consequence that far less is known about the specific termination sites of these projections within prefrontal cortex. This shortcoming is particularly noticeable for our understanding of the efferents from the hippocampus. Consequently, the extent and nature of any convergence between the hippocampus and amygdala within the primate prefrontal cortex remains poorly understood. Such convergence is of growing interest. There is, for example, increasing acceptance that the anterior hippocampus has functions related to stress and affect (Fanselow and Dong 2010; Strange et al. 2014), which could complement those of the amygdala (Roozendaal et al. 2009) through their prefrontal connections. In addition, the amygdala can facilitate the ways in which emotions influence autobiographical memory (McGaugh 2000; Talarico et al. 2004), a function thought to involve interactions with the hippocampus and prefrontal cortex (Fink et al. 1996; LaBar and Cabeza 2006).

To visualize termination sites, it is necessary to use anterograde tracers. To date, the sole anterograde tracer study of monkey hippocampal efferents provided only summary data, with no detailed area or lamina information (Rosene and Van Hoesen 1977). A key goal was, therefore, to detail the termination pattern of hippocampal efferents within the prefrontal cortex. An important aspect was to place tracers along the anterior–posterior length of the hippocampus, given the evidence for changing functions in this dimension (Fanselow and Dong 2010; Aggleton 2012; Strange et al. 2014). The prefrontal projections from the amygdala have been described more fully using anterograde tracers (Porrino et al. 1981; Amaral and Price 1984; Ghashgaei et al. 2007). Relatively dense amygdala projections terminate throughout areas 24, 25, and 32 on the medial surface and along areas 12 and 14 on the orbital surface. Lighter projections to the dorsolateral and ventrolateral surfaces are seen in parts of areas 6, 45, 46, and lateral 12 (Amaral and Price 1984; Ghashgaei et al. 2007). Particularly striking is evidence from a study using biotinylated dextran amine (BDA) that the amygdala projects to almost all prefrontal areas, with varying degrees of density (Ghashgaei et al. 2007). The present study sought to confirm and extend these amygdala findings. Key features of the amygdala experiments include the number of tracer injections targeting individual amygdala nuclei, along with the use of more fine-grained distinctions within prefrontal areas than reported by Ghashgaei et al. (2007). By combining both amygdala and hippocampal projection data in one study, it was also possible to provide direct comparisons between their prefrontal inputs.

The injections of anterograde tracers within the amygdala largely targeted the basal nuclei, which retrograde tracer studies show to be the principal source of the prefrontal inputs from this structure (Jacobson and Trojanowski 1975; Morecraft et al. 1992; Carmichael and Price 1995). Likewise, retrograde tracer studies have shown that within the hippocampus the direct prefrontal inputs arise from the subiculum and immediately adjacent parts of CA1 (Morecraft et al. 1992; Barbas and Blatt 1995; Carmichael and Price 1995; Insausti and Munoz 2001). Attention, therefore, focused on those cases with injections in one or both of these hippocampal areas. An additional goal was to determine whether the hippocampal projections to the prefrontal cortex rely solely on the fornix. While fornical fibers can be followed to prefrontal areas (Poletti and Cresswell 1977), it remains uncertain whether there are alternate, direct routes from the hippocampus. This is a potentially important question as fornix damage has repeatedly been used in both monkeys and humans to explore hippocampal processing. Consequently, a subset of macaque monkeys that had received fornix transections, principally for the purpose of behavioral studies, also received anterograde tracer injections in the hippocampus.

## Materials and Methods

The data in this study were taken from 2 cohorts of monkeys from different research centers [Laboratory of Neuropsychology, National Institute of Mental Health (NIMH) and the Department of Neurobiology and Anatomy, Boston University, School of Medicine]. The purpose was to maximize available information. The NIMH cohort comprised 17 adult cynomolgus monkeys (*Macaca fascicularis*) and 1 rhesus monkey (*Macaca mulatta*). The Boston University cohort comprised 9 adult rhesus monkeys. In both cohorts, radioactive amino acids had been injected into the medial temporal lobe. In a number of cases, these injections were bilateral. Despite some minor variations in methodology, as well as the use of 2 closely related macaque species, there was a very clear consistency across the resulting findings. All experimental procedures were conducted consistent with the NIH Guide for Care and Use of Laboratory Animals (NIH Publication No. 86–23, revised 1985).

### General Surgical Procedures

NIMH cohort: Prior to the amino acid injections, all animals were lightly sedated with ketamine hydrochloride (10 mg/kg), deeply anesthetized with sodium pentobarbital (35 mg/kg), and placed in a stereotaxic apparatus. Under aseptic conditions, bone and dural flaps were opened to permit access to the temporal lobe. Following injection of the tracer, the dura and skin were sutured in anatomical layers. Immediately following surgery, as each animal began to wake, it was placed in a heated recovery cage in which humidity and oxygen levels were controlled. In all cases, recovery was without incident. Prophylactic doses of antibiotics were administered to prevent infection (Bicillin, Wyeth Laboratories) whereas dexamethasone phosphate (0.3 mg/kg) was given immediately after surgery to reduce any cerebral edema. The analgesic morphine (1 to 2 mg/kg subcutaneous every 4 h) was given according to NIMH veterinary guidance. Recovery was without incident. After an interval of 6 or 7 days, the monkeys were deeply anesthetized with an overdose of sodium pentobarbital (100 mg/kg i.v.) and transcardially perfused with normal saline followed by neutral buffered formalin.

Boston University cohort: Each animal was lightly sedated with ketamine hydrochloride (10–15 mg/kg) and deeply anesthetized by intravenous sodium pentobarbital (35 mg/kg). The surgery was performed under aseptic conditions and at its completion the wound was closed in anatomical layers so that the dura, muscle and skin were sutured. Prophylactic doses of Bicillin were given and analgesics provided (Banamine IM, 1.0 mg/kg). Analgesia was continued for 48–96 h, or longer if needed, as determined by veterinary staff. Other surgical procedures matched those described for the NIMH cohort. Following a survival period of 5 to 10 days, the animals were deeply anesthetized with sodium pentobarbital and transcardially perfused with 4% paraformaldehyde.

### Amygdala Injections

All of these cases, which were from the NIMH cohort, have been included in other studies (e.g., Aggleton and Mishkin 1984). Each animal received an injection of an equal-parts mixture of tritiated proline (New England Nuclear, L-[2, 3, 4, 5 H], specific activity 139 Ci/mmole) and leucine (New England Nuclear L-[3, 4, 5 H], specific activity 111 Ci/mmole). Injections were made through a 1-µl Hamilton syringe at a final concentration of 50 µCi/µl. Single injections of between 0.1 and 0.2 µl of the radioactive H^3^ amino acid mixture (i.e., 5–10 μCi) were made in 8 cynomolgus monkeys. A pair of injections (0.20 and 0.30 µl, total 50 μCi) was, however, made in the basal nucleus in the same hemisphere in the ninth monkey (ACy6). All injections were via a dorsal stereotaxic approach. Injection coordinates were derived from skull landmarks revealed on X rays (Aggleton and Passingham 1981). Six of these monkeys received unilateral injections (ACy6, ACy10, ACy13, ACy16, ACy17, and ACy18) whereas 3 received bilateral injections (ACy20, ACy21, and ACy22), making a total of 12 injection sites. Following perfusion, the brains were cryoprotected with 30% sucrose solution prior to being cut into 33-µm coronal sections on a freezing microtome. Every sixth section was mounted on a glass slide from either phosphate buffer or Perfix and then coated with Kodak NTB2 emulsion. The sections were exposed at 4°C for 6–30 weeks, developed in Kodak Dl9, fixed, and counterstained with thionine. For each animal, there was series with a minimum exposure duration of 12 weeks.

### Hippocampal Injections

The data came from 2 closely related studies. The NIMH cohort contained 7 cynomolgus monkeys (all designated “ACy”) and 1 rhesus monkey (ARhF24). These cases have been described in other studies (e.g., Aggleton et al. 1986). The surgical procedure was essentially the same as that described for the amygdala injections so that each injection was an equal-parts mixture of tritiated proline and leucine at a final concentration of 50 µCi/µl. A single injection ranging from 0.10 to 0.20 µl (5–10 μCi) was made in 4 cases (ACy12, ACy14, ACyF15, and ACyF19), whereas 2 monkeys (ACy25 and ACy28) received multiple injections totaling from 0.24 to 0.44 µl (12 and 22 μCi, respectively) within the same hemisphere. In 1 further case (ACyF27), injections were placed in both hemispheres. In the left hemisphere, a single injection was centered in the caudal subiculum (ACyF27L, 6μCi), whereas a pair of injections in the right hemisphere involved the rostral presubiculum and caudal perirhinal cortex, as well as the subiculum (ACyF27R, total 20.5μCi). The coordinates for the hippocampal injections were determined with the aid of electrophysiological recordings made immediately prior to the injection with a tungsten microelectrode (Aggleton et al. 1986). Tissue perfusion and treatment of the sections was identical to that described for the animals with amygdala injections.

Four of the 8 monkeys from the NIMH group had previously received surgical transections of the fornix 2–12 months prior to the injection of the amino acids (all such cases are labeled either ACyF or ARhF). The fornix surgeries were principally conducted for behavioral studies whereas the surgical procedures and the completeness of the lesions have been documented elsewhere (Bachevalier et al. 1985). Fornix transection does not result in overt cell loss in the hippocampal formation (Daitz and Powell 1954), and it has been shown that those hippocampal cells that have axons cut due to the fornix surgery still remain capable of transporting amino acids after surgery (Aggleton et al. 1986; Saunders and Aggleton 2007).

The hippocampal study also included the cohort of 9 rhesus monkeys from Boston University. Each case received a single tracer injection per hemisphere (sometimes an additional injection was made in the opposite hemisphere). Each injection contained a mixture of tritiated leucine, lysine, and proline, usually derived from an algal protein hydrolysate (Saunders and Rosene 1988). Stock solutions of the amino acid mixture were desiccated under gaseous nitrogen and reconstituted with sterile saline at a concentration of 100µCi/µl. Stereotaxic injections, which ranged between 15 and 50 μCi, were made via an injection electrode attached to a 5-µl Hamilton syringe (see Saunders and Rosene 1988). Other surgical procedures matched those already described. Following perfusion, the brains were stored in 10% formalin for 2 weeks, then embedded in paraffin, and cut into 10-µm coronal sections. Sections were mounted on glass slides coated with Kodak NTB2 emulsion, stored at 4°C in the dark, and subsequently processed using a method modified from Cowan et al. (1971). For each animal, there was more than 1 series of sections. Each individual series was stored for between 6 and 12 weeks prior to development and subsequent Nissl staining. Some of these cases have been described previously (Rosene and Van Hoesen 1977; Blatt and Rosene 1998).

While all available sections were examined in both darkfield and brightfield, e.g., to compile Tables 1–3, only a subset of sections have been plotted in detail. Other sections (from the NIMH monkeys) were copied, though in less detail, close to the time of the tracer injections. The coronal sections plotted in detail were approximately equidistant and contained all major prefrontal areas. Two independent observers (JPA and NFW) made decisions concerning label density. Variations in injection volume and concentration, imaging time and section thickness (in the 2 hippocampal cohorts) limited any quantitative comparisons between cases.

**Table 1:** Distribution of label on the orbital surface of the frontal lobe

Site	Code	10o	11m	11l	13b	13m	13l	13a*	Iam*	Iai*	Ial*	Iapm*	Iapl*	G
A Bi/mc	ACy21L				i II	i II VI	i II VI		VI	*I II* III V *VI*	*i II iii VI*	*VI*	*i II* iii VI	I *II*
A Bi/mc	ACy21R			i II	i II		i II VI	i II	VI	*I II* III V *VI*	*i II* iii *VI*	*VI*	*i II* iii	*I II*
A Bi/mc	ACy6		i II			i II			VI	*I II III V* VI	*i* *II*	*III* *VI*	*I-VI*	I
A AB	ACy20L									V-VI	V-VI	V-VI		
A AB	ACy20R									V-VI	V-VI	V-VI		
A AB	ACy18									V-VI	V-VI	V-VI		
A Bpc	ACy10				V-VI				V-VI	V-VI	V-VI	V-VI	V-VI	
A L	ACy16								I					
ACe/Bmc	ACy17					I	I	I	I	I	I	I	I	
SubA	ACy12		III		III			III						
SubA	ACy14				III			III						
Sub/CA1M	MLPl				III	III								
Sub/CA1M	MRC				III			III						
Sub/CA1P	ACy28		III		III	III		III						

Note: The Roman numerals refer to the lamina of termination. Underline type shows where label appears most dense. A blank indicates no observed label. Sites with an asterisk lack a granular layer IV. For the amygdala projections, some cases displayed label confined to the deepest level of layer I or the most superficial level of layer III. These instances are shown by a smaller font. The “Site” column refers to the location of the injection, so that injections in the amygdala are designated A, those in the hippocampus are Sub (subiculum) and/or the hippocampal CA fields. For the amygdala injections, the other letters refer to nuclei (AB, accessory basal; Bi, intermediate division of basal nucleus; Bmc, magnocellular division of the basal nucleus; Bpc, parvicellular division of the basal nucleus; Ce, central; L, lateral). For the hippocampus, the final letter refers to the anterior–posterior level of the injection (A, anterior M, mid; P, posterior).

**Table 2:** Distribution of label on the medial surface of the frontal lobe

Site	Code	10m	14r	14c*	32*	24a*	24b*	24c*	25*
A Bi/mc	ACy21L	(II)	i II	*I II* VI	*i II* iii *V VI*	*i II* iii *VI*	*i II* iii *V VI*	*i II* iii VI	*I* II-VI
A Bi/mc	ACy21R	(II)		*I II* VI	*i II* iii *V VI*	*i II* iii VI	i *II* iii V VI	i *II* iii VI	*I* II-VI
A Bi/mc	ACy6			I VI	i II iii	i II iii	i II iii VI	II i iii VI	I
A AB	ACy20L								I
A AB	ACy20R								I
A AB	ACy18								I
A Bpc	ACy10			V-VI	II-VI		II iii-iv		
A L	ACy16								I-VI
ACe/Bmc	ACy17			*I*					*I*
SubA	ACy12		III	*II-VI*	III				*II-VI*
SubA	ACy14		III	II-VI	III	III			*II* iii-iv
Sub/CA1M	MLP-L			III-VI		III			II-VI
Sub/CA1M	MRC			III					
Sub/CA1–4P	ACy28		III	*III-VI*		III-V			I-VI

Note: The Roman numerals refer to the lamina of termination. Underline type shows where the label appears most dense. A blank indicates no observed label. Sites with an asterisk lack a granular layer IV. In area 25, layers II and III are largely the same, as are layers V and VI. For the amygdala projections, some cases displayed label confined to the deepest level of layer I or the most superficial level of layer III. These instances are shown by a smaller font. The “Site” column refers to the location of the injection, so that injections in the amygdala are designated A, those in the hippocampus are Sub (subiculum) and/or the hippocampal CA fields. For the amygdala injections, the other letters refer to nuclei (AB, accessory basal; B, basal; Ce, central; L, lateral; Bi, intermediate division of basal nucleus; Bmc, magnocellular division of the basal nucleus; Bpc, parvicellular division of basal nucleus). For the hippocampus, the final letter refers to the anterior–posterior location of the injection site (A, anterior M, mid; P, posterior). The inputs to area 10m are in parenthesis as they are restricted to the caudal limit of the area.

**Table 3:** Distribution of label on the lateral surface of the frontal lobe

Site	Code	9m	9l	8	46	45	6d	6v	12l	12o	12m	12r	PrCo
A Bi/mc	ACy21L	i *II* VI			i II	i *II* iii *VI*	i *II*	i *II* VI	i *II* VI	i *II* VI	i *II*		i *II* iii VI
A Bi/mc	ACy21R	i *II* VI	i II		i II	i *II* iii *VI*	i II VI	i *II VI*	i II VI	i II VI	i II		i *II* iii VI
A Bi/mc	ACy6	i II	i II VI			i *II* iii *VI*	i II	i II VI	i II VI	i *II*	i II VI	i II	i *II* III
A AB	ACy20L												
A AB	ACy20R												
A AB	ACy18												
A Bpc	ACy10												
A L	ACy16												
ACe/Bmc	ACy17												

Note: The Roman numerals refer to the lamina of termination. For the amygdala projections, some cases displayed label confined to the deepest level of layer I or the most superficial level of layer III. These instances are shown by a smaller font. Underline type shows where the label appears most dense. A blank indicates no observed label. The “Site” column refers to the location of the injection, so that injections in the amygdala are designated A whereas the other letters refer to nuclei (AB, accessory basal; Bi, intermediate division of basal nucleus; Bmc, magnocellular division of the basal nucleus; Bpc, parvicellular division of the basal nucleus; Ce, central; L, lateral). The hippocampal injection cases are not included as there was no evidence of a projection to this region.

### Nomenclature

The designation of the various amygdala nuclei follows that of Amaral et al. (1992). One consequence is that the basal nucleus is divided into several subregions. The parvicellular division of the basal nucleus largely corresponds to the medial basal nucleus of Crosby and Humphrey (1941), whereas the magnocellular and intermediate divisions of the basal nucleus correspond to the lateral basal nucleus (Crosby and Humphrey 1941). The intermediate division of the basal nucleus forms the region between the parvicellular and magnocellular divisions (Amaral et al. 1992; Friedman et al. 2002). There is a separate accessory basal nucleus (Crosby and Humphrey 1941; Amaral et al. 1992).

The designations of the various hippocampal subfields and adjacent regions closely follow the descriptions of Lorente de Nó (1934), which have been widely adopted for the monkey brain. Consequently, the term prosubiculum refers to the transition area between CA1 and the subiculum (Lorente de Nó 1934; Saunders and Rosene 1988; Ding 2013). Distal to the subiculum are found the presubiculum and parasubiculum (Lorente de Nó 1934; Saunders and Rosene 1988; Ding 2013). The terms “proximal” and “distal” refer to locations within the hippocampal formation, with respect to whether they are near (“proximal”) or far (“distal”) from the dentate gyrus, assuming the hippocampus was to be unrolled flat. Consequently, distal CA1 is close to the subiculum border, proximal subiculum, i.e., prosubiculum, is adjacent to CA1, whereas the distal subiculum is adjacent to the presubiculum (see van Strien et al. 2009).

The subfields within the medial and orbital prefrontal cortex match those described by Carmichael and Price (1994). Their designations were largely based on those of Walker (1940), but Carmichael and Price (1994) described additional subfields, and some borders have moved appreciably, for example, area 10 has been extended and subdivided into 5 areas. A consequence is that the majority of prefrontal areas is numbered (Walker 1940) but has often been further subdivided using letters that often help to locate the subarea (Carmichael and Price 1994). The letter “r” refers to rostral (areas 14r and 12r) whereas “c” refers to caudal (area 14c), except for area 24, which has traditionally been divided into areas 24a, 24b, and 24c, going increasingly dorsal above the corpus callosum (Vogt et al. 2005). The letter “m” refers to medial (areas 9m, 10m, 11m, 12m, and 13m) whereas “l” refers to lateral (areas 9l, 11l, 12l, and 13l). Area 6 is divided into dorsal (6d) and ventral (6v) portions. Other designations concern the caudal part of area 12 (area 12o), whereas the medial part of area 13, which had previously been included within area 14 (Walker 1940), has been split into rostral (area 13b) and caudal (area 13a) components (Carmichael and Price 1994). The agranular insula (Ia) is divided into anterior medial (Iam), anterior intermediate (Iai), anterior lateral (Ial), posterior medial (Iapm), and posterior lateral (Iapl) subdivisions (Carmichael and Price 1994). Ventral to the gustatory cortex (G) in the sylvian fissure are the dysgranular (Id) and agranular (Ia) parts of the temporal insula.

The terminology used by Carmichael and Price (1994) has the particular merit that previous descriptions of amygdala and hippocampal projections to this region using anterograde and retrograde tracers, respectively, largely used the same scheme (Amaral and Price 1984; Carmichael and Price 1995). For this reason, later designations (e.g., Paxinos et al. 2009) have not been incorporated. The nomenclature for the lateral prefrontal cortex is also based on Walker (1940), but later modified by Amaral and Price (1984). Additional refinements for the lateral and premotor cortex designations were taken from Carmichael and Price (1995). Many of the borders in the prefrontal cortex are, however, indistinct and there can be extensive transitional areas. Consequently, it can be difficult to demarcate some borders in Nissl-stained sections (Carmichael and Price 1994). Finally, the prefrontal nomenclature used by Ghashgaei et al. (2007) in their study of amygdala efferents sometimes differs from that of Carmichael and Price (1994). Where possible, the terminology for the different prefrontal areas used by Ghashgaei et al. (2007) is converted to that of Carmichael and Price (1994).

## Results

The study combined archival data to remove the need for new monkey cases. This approach was made possible by the fact that the technique used to visualize the tracer (autoradiography) is exceptionally stable. Comparisons based on old photomicrographs confirm this assumption.

### Amygdala Projections

**Figure 1.**
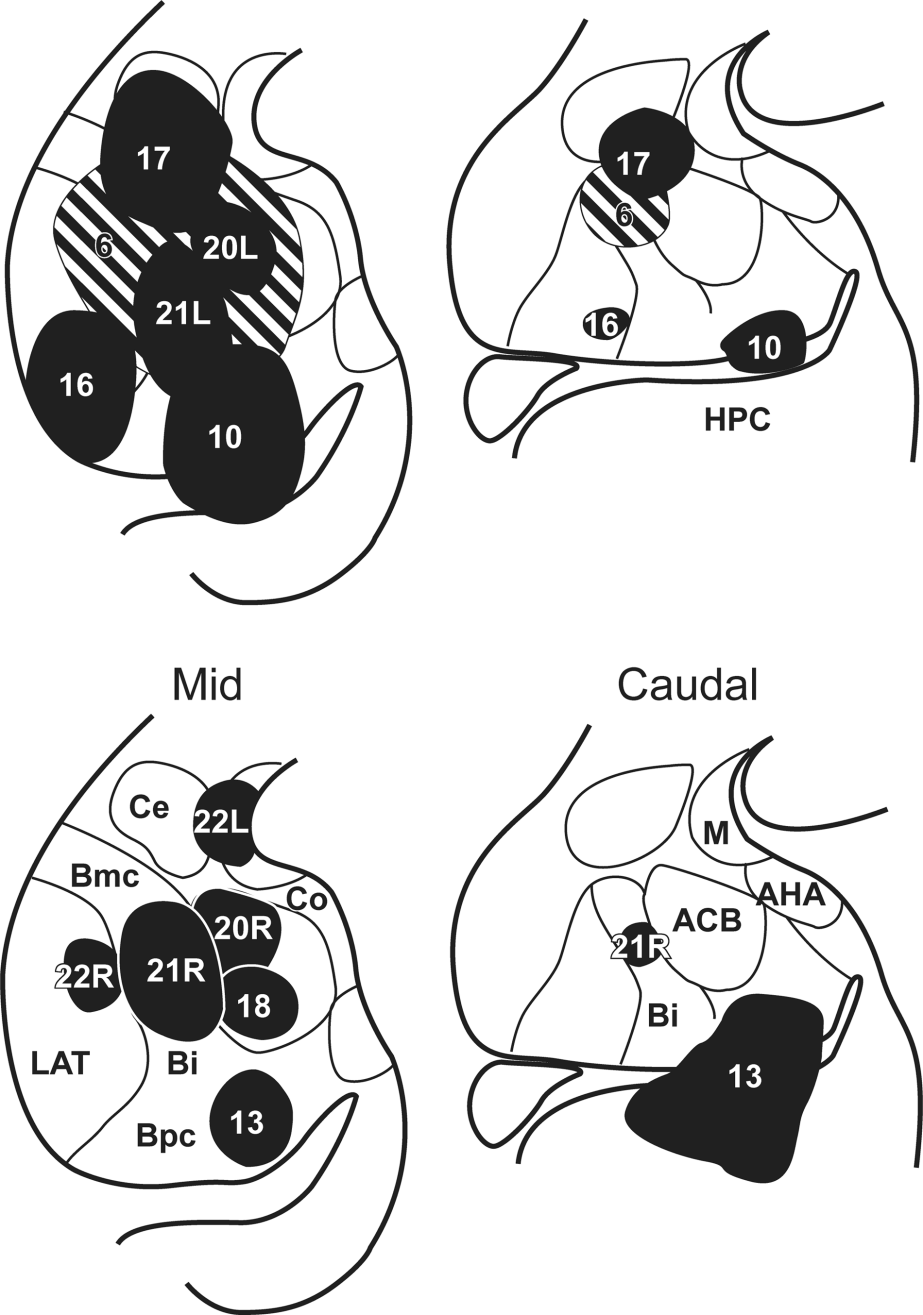
Location and extent of the amino acid injections into the amygdala. All cases are depicted. The numbers correspond to the injection cases, where L and R refer to the left and right hemispheres of those cases with injections in both hemispheres. The injection sites are depicted on standard coronal sections at the level of the middle, with those that extended more posteriorly also shown on an additional section in the posterior third of the amygdala. ACB, accessory basal nucleus; AHA, amygdalo-hippocampal area; Bi, basal nucleus, intermediate division; Bmc, basal nucleus, magnocellular division; Bpc, basal nucleus, parvicellular division; Ce, central nucleus; Co, cortical nucleus; HPC, hippocampus; LAT, lateral nucleus; M, medial nucleus.

**Figure 2.**
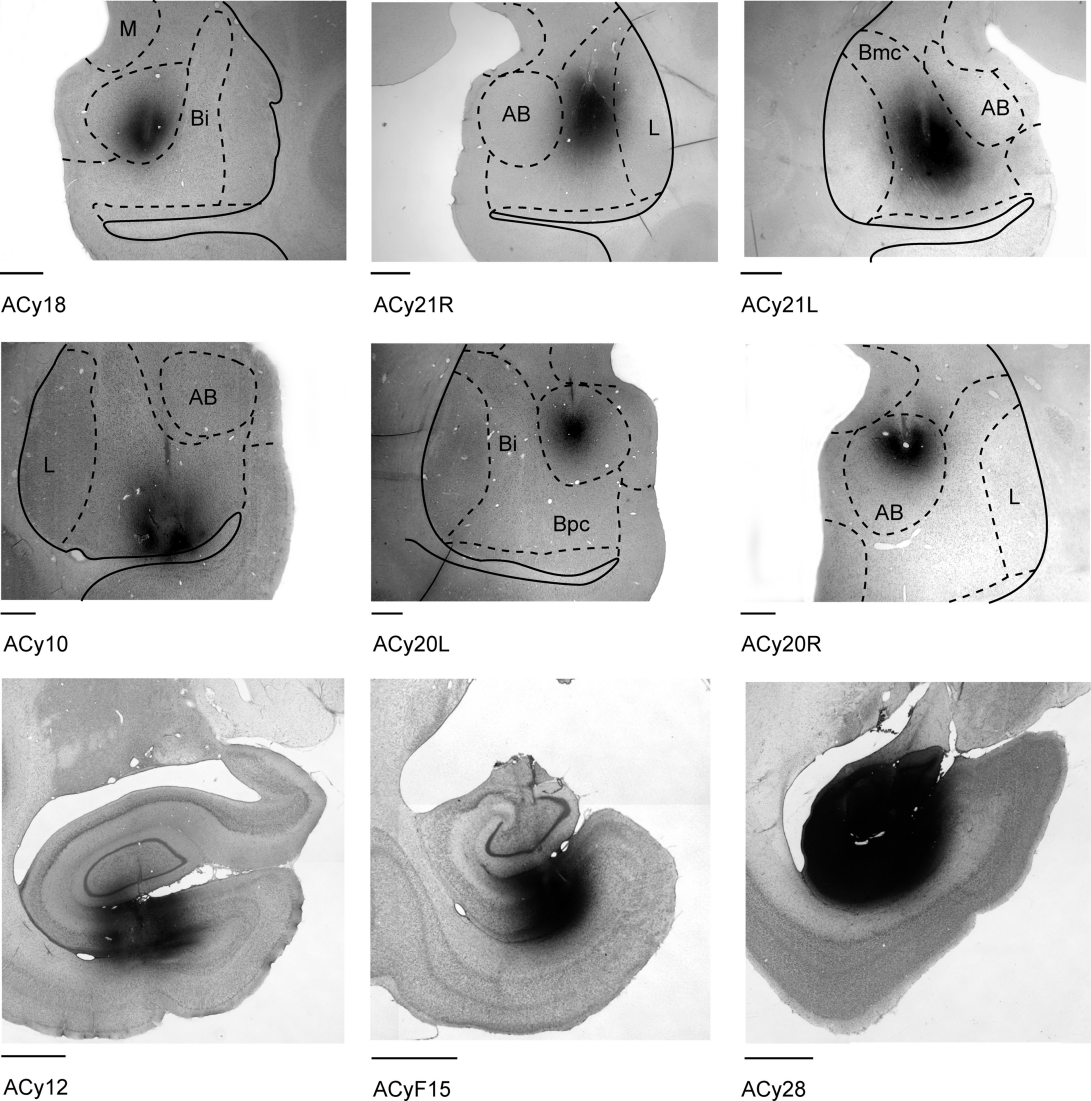
Brightfield coronal photomicrographs showing the center of the amino acid injection sites in 6 amygdala cases (top 2 rows) and 3 hippocampal cases (bottom row). These cases were selected as they illustrate how different amygdala nuclei and different anterior–posterior levels in the hippocampus were targeted. Abbreviations are as in Figure 1. The scale bar corresponds to 1.0 mm.

Figures 1 and 2 depict the placement and extent of all of the injections of tritiated amino acids into the amygdala included in this study. The effective injection site is considered as the area in which silver grains filled the neuropil and perikarya at a density that was appreciably above background. The intention was to place injections in the center of each nucleus, to avoid spread into adjacent structures. For this reason, the surgeries targeted the midlevel of the amygdala (Fig. 2). While this approach made it possible to locate separate injections in all of the basal nuclei (Fig. 1), some of the most anterior and posterior portions of the amygdala were not covered.

It was immediately apparent that there were striking differences in the extent of the prefrontal label from the various injection sites. Only those injections placed in the intermediate and magnocellular parts of the basal nucleus (the lateral basal nucleus) resulted in widespread prefrontal label, which was found across orbital, medial, and lateral areas (cases ACy21L, ACy21R, and ACy6). The 2 injections in monkey ACy21 were of similar extent, and both were centered in the intermediate division of the basal nucleus in different hemispheres (Fig. 2) but extended dorsally to reach the ventral part of the magnocellular division. As the results from case ACy6 (see below) show that few, if any, prefrontal projections from this area cross to the contralateral hemisphere, the 2 injections in monkey ACy21 are treated as essentially independent.

**Figure 3.**
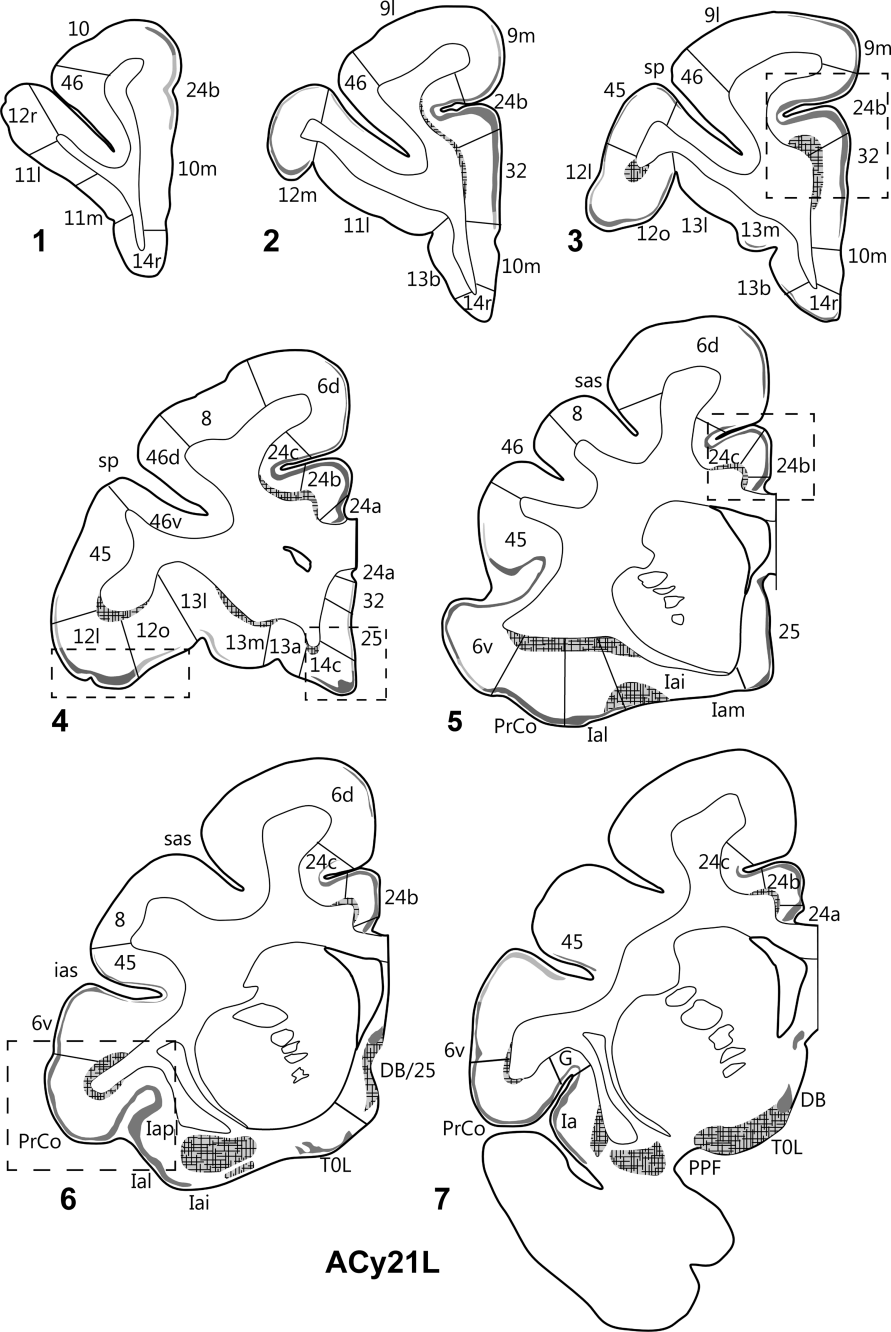
Projections from the intermediate and magnocellular parts of the basal amygdala nucleus. Series of drawings of coronal sections from case ACy21L going from anterior (#1) to posterior (#7). The numbers (letters in the case of the insula) correspond to different prefrontal areas. Terminal label is shown in gray, with darker gray representing denser label. The cross-hatching marks those areas with both fiber and terminal labeling. The boxes in dashed lines show the regions in the darkfield images in Figures 5 and 6. DB, diagonal band; DB/25, transition zone between the diagonal band and area 25; G, gustatory area; ias, inferior arcuate sulcus; PPF, prepiriform cortex; PrCo, precentral opercular area; sas, superior arcuate sulcus; sp, sulcus principalis; TOL, olfactory tubercle.

**Figure 4.**
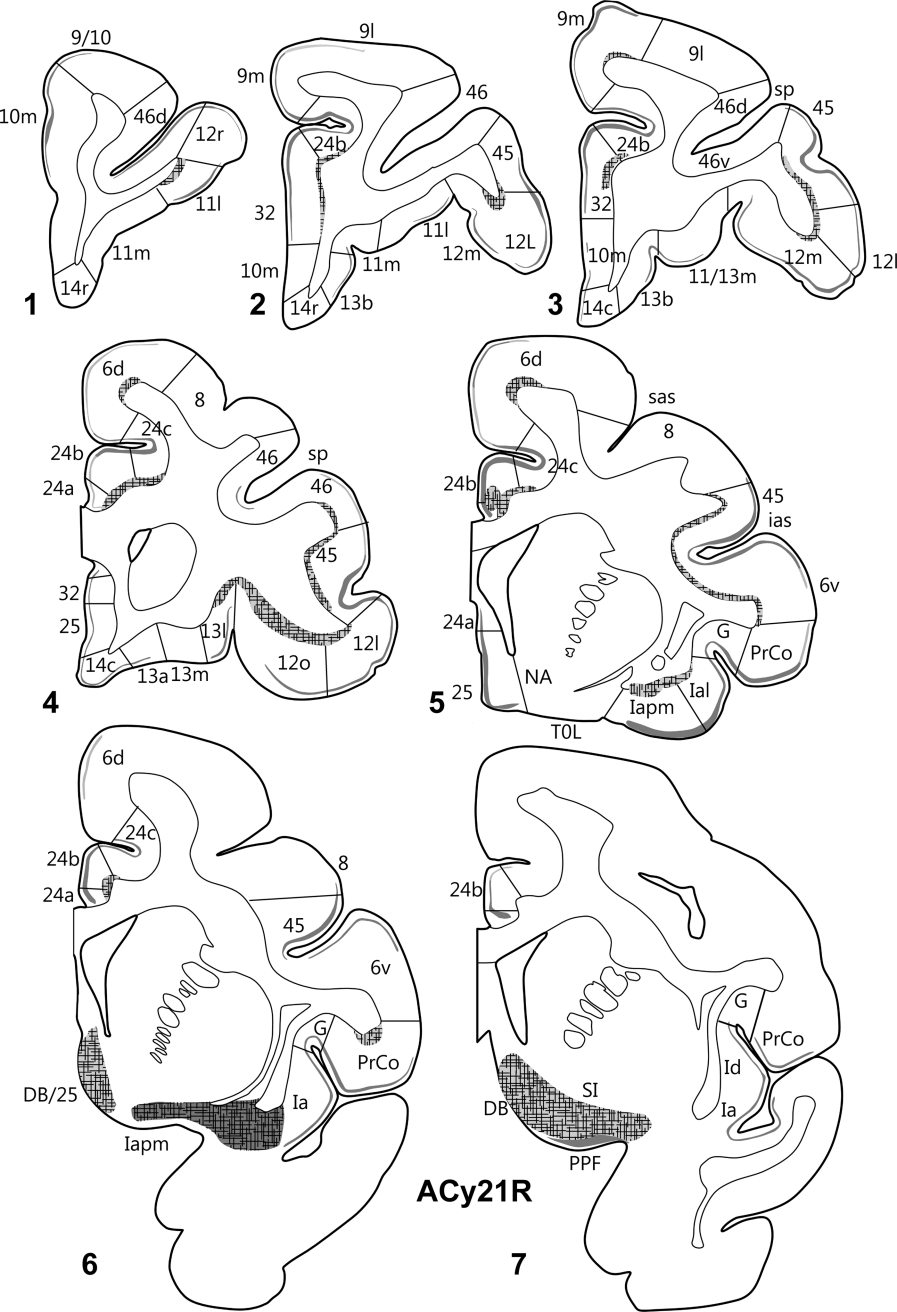
Projections from the intermediate and magnocellular parts of the basal amygdala nucleus. Series of drawings of coronal sections from case ACy21R going from anterior (#1) to posterior (#7). The numbers (letters in the case of the insula) correspond to different prefrontal areas. Terminal label is shown in gray, with darker gray representing denser label. The cross-hatching marks those areas with both fiber and terminal labeling. SI, substantia innominata; all other abbreviations are as in Figure 3.

**Figure 5.**
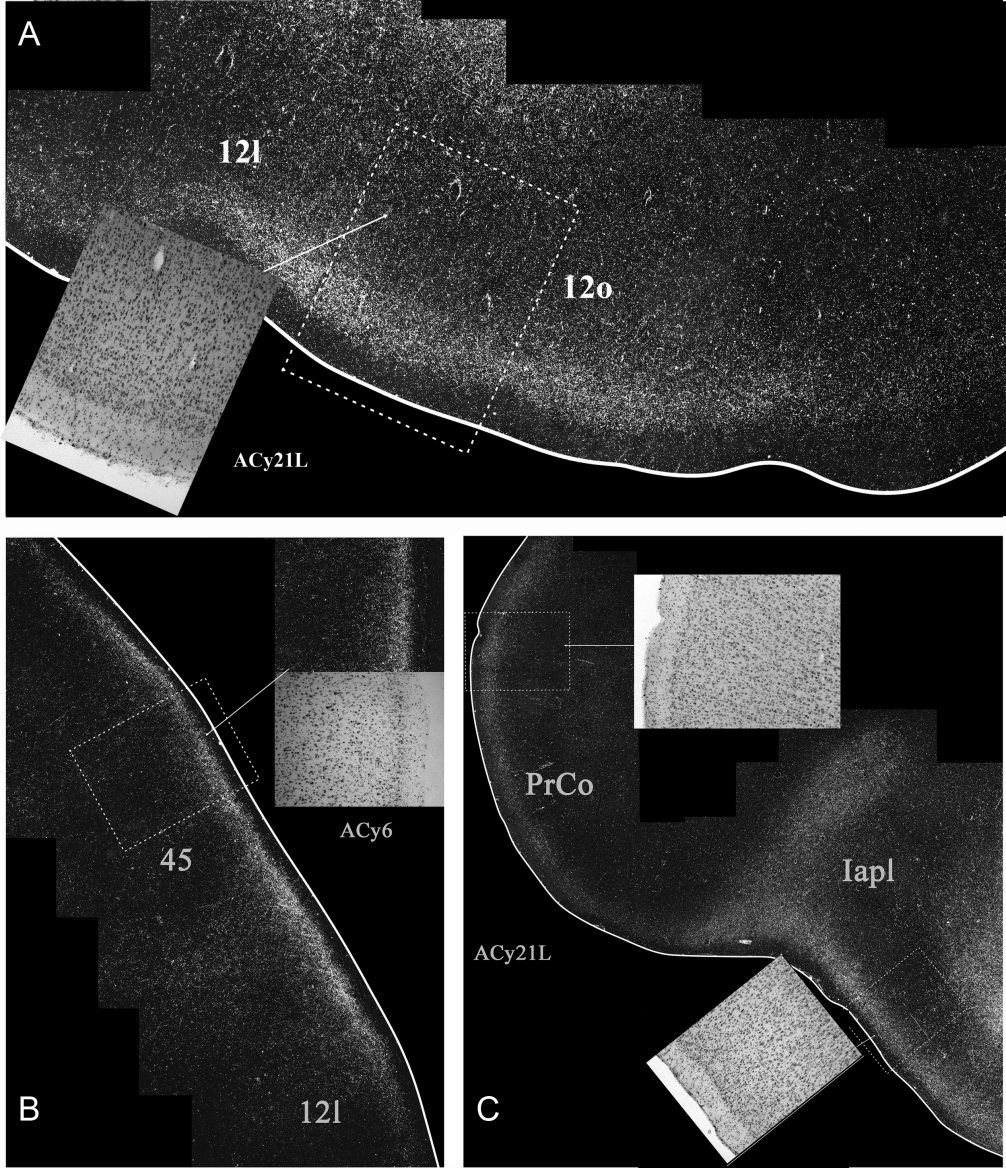
Darkfield images of autoradiographic label in 2 cases (ACy21L and ACy6), both with injections in the intermediate and magnocellular parts of the basal amygdala nucleus. The images are of the junction of the orbital and lateral prefrontal cortex (*A*,*C*) and that of the lateral prefrontal cortex (*B*). The brightfield images correspond to the subregions marked by boxes with dashed lines. The entire area of each darkfield image is indicated in Figures 3 and 7.

The 2 hemispheres of monkey ACy21 (Figs 3 and 4) are described concurrently, with the label in the orbital (Table 1), medial (Table 2), and then lateral (Table 3) surfaces of the prefrontal cortex reported in that order. On the orbital surface, the most rostral label was in area 11l (deep I and II), but this label was only found in case ACy21R (Fig. 4). Both cases contained light, but variable, terminal label in area 13. In case ACy21R, this label was most evident in 13l, whereas in the opposite hemisphere (ACy21L), the area 13 label was most evident in 13m, where it sometimes extended into 13l. In both hemispheres, some label was also present in 13b (Figs 3,4). In case ACy21L, the area 13b label was continuous with label in adjacent area 14r. The projections to area 13 consistently terminated in deep layer I and layer II, whereas additional label was present in layer VI of area 13l. More caudally, considerable label was found across the agranular insula, which included many fibers in the deeper layers. The insula label, which was most dense in areas Ial, Iapm, Iai, and Iapl, continued laterally into the gustatory cortex as well as into PrCo (precentral opercular cortex, Fig. 5C). The label in areas Iai, Ial, and Iapl was particularly dense in layers I and II, whereas in areas Iam and Iapm the label, much of it fibers, was concentrated in the deepest layer (Table 1).

**Figure 6.**
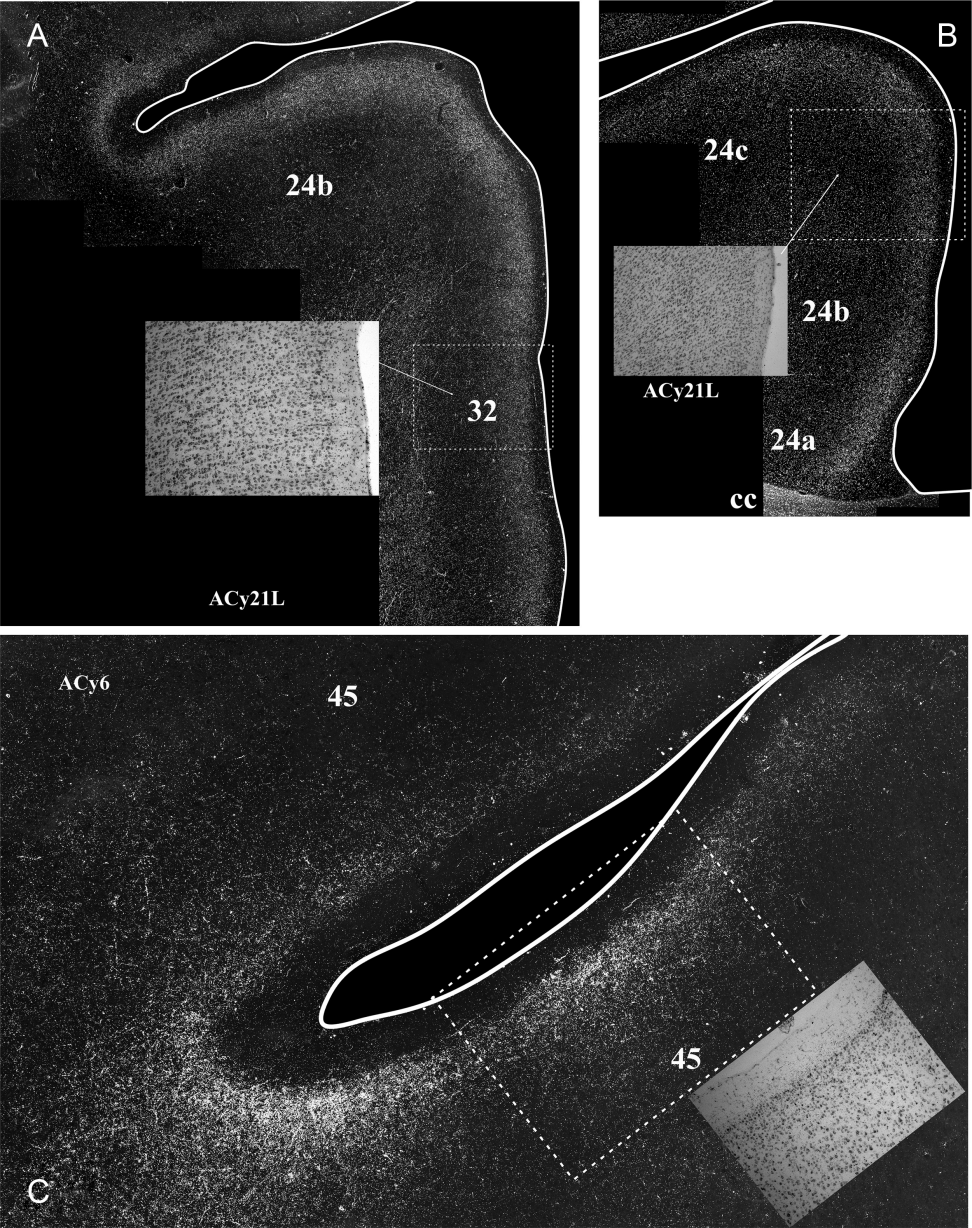
Darkfield images of autoradiographic label in 2 cases (ACy21L and ACy6), both with injections in the intermediate and magnocellular parts of the basal amygdala nucleus. The images are of the anterior cingulate cortex (*A*,*B*) and the inferior arcuate sulcus (*C*). The brightfield images correspond to the subregions marked by boxes with dashed lines. The entire area of each darkfield image is indicated in Figures 3 and 7. cc, corpus callosum.

On the medial surface of cases ACy21R and ACy21L (Figs 3,4), label was absent from the frontal pole (rostral area 10). Instead, the most anterior label in the medial wall was at the transition between area 10m (layer II) and area 24b (ACy21L, Fig. 3). At slightly more posterior levels, additional label was found in area 32. The label in areas 24b and dorsal 32 at this pregenual level was particularly dense (Fig. 6*A*). At the genu of the corpus callosum, a continuous line of very dense label was seen in the medial wall that started ventrally in area 32 and extended dorsally through areas 24a, 24b, and 24c (the label in area 24c being most dense close to area 24b). The label continued more lightly into area 6d on the medial wall and dorsal convexity. The label in areas 24 and 32 was concentrated in deep I, layer II, and superficial III, along with a mixture of fibers and termination in layers V and VI. Above the corpus callosum, the area 24 label showed a ventral–dorsal gradient (most dense in 24a, least in 24c, Fig. 6*C*), with the labeling continuing along the depth of the lower bank of the cingulate sulcus. By the level of the anterior thalamus, the label in 24b and 24c had almost vanished, leaving the label largely confined to 24a. Above the thalamus, the area 24a label continued to diminish going caudally, where it ceased a little before the appearance of the retrosplenial cortex. Below the genu, frontal label was consistently found in areas 24a, 32, 25, and 14c. The label in area 14c (layers I and II, with lighter label in VI) continued forward to the border with 14r, where it rapidly diminished and disappeared. The label in area 25 (subcallosal) was striking as there were regions with densely labeled fibers that ran just deep to area 25, along with many fibers that passed through area 25. At its mid-AP level, the area 25 label was concentrated in layer I, but at the caudal limits of area 25, label was found across all cortical layers, reflecting fibers and possible termination (Table 2).

Some of the most anterior lateral in case ACy21 was found in area 9m, where the label continued from the medial wall dorsally onto the most medial part of the dorsal surface (area 9l) (Figs 3,4). At the same level, isolated patches of label were present in area 46 in the lower bank of sulcus principalis, sometimes accompanied by label in area 45 (Figs 3,4). A little more caudal, very clear terminal label was found across area 12 in deep I, II, and layer VI (Fig. 5*A*). The dense label in area 12l continued onto the orbital surface (12m and 12o). The label in area 12l also continued dorsally to involve much of area 45 (especially case ACy21R, see also Fig. 5*B*). At its dorsal margin, this label in area 45 sometimes extended into area 46 (case ACy21R, only light label). More posterior, the label in area 45 became continuous with more ventral label in area 6v and the precentral opercular cortex (PrCO), the latter label being particularly dense (Fig. 5*C*). At its posterior and dorsal limit, the label in area 45 reached the border with area 8.

**Figure 7.**
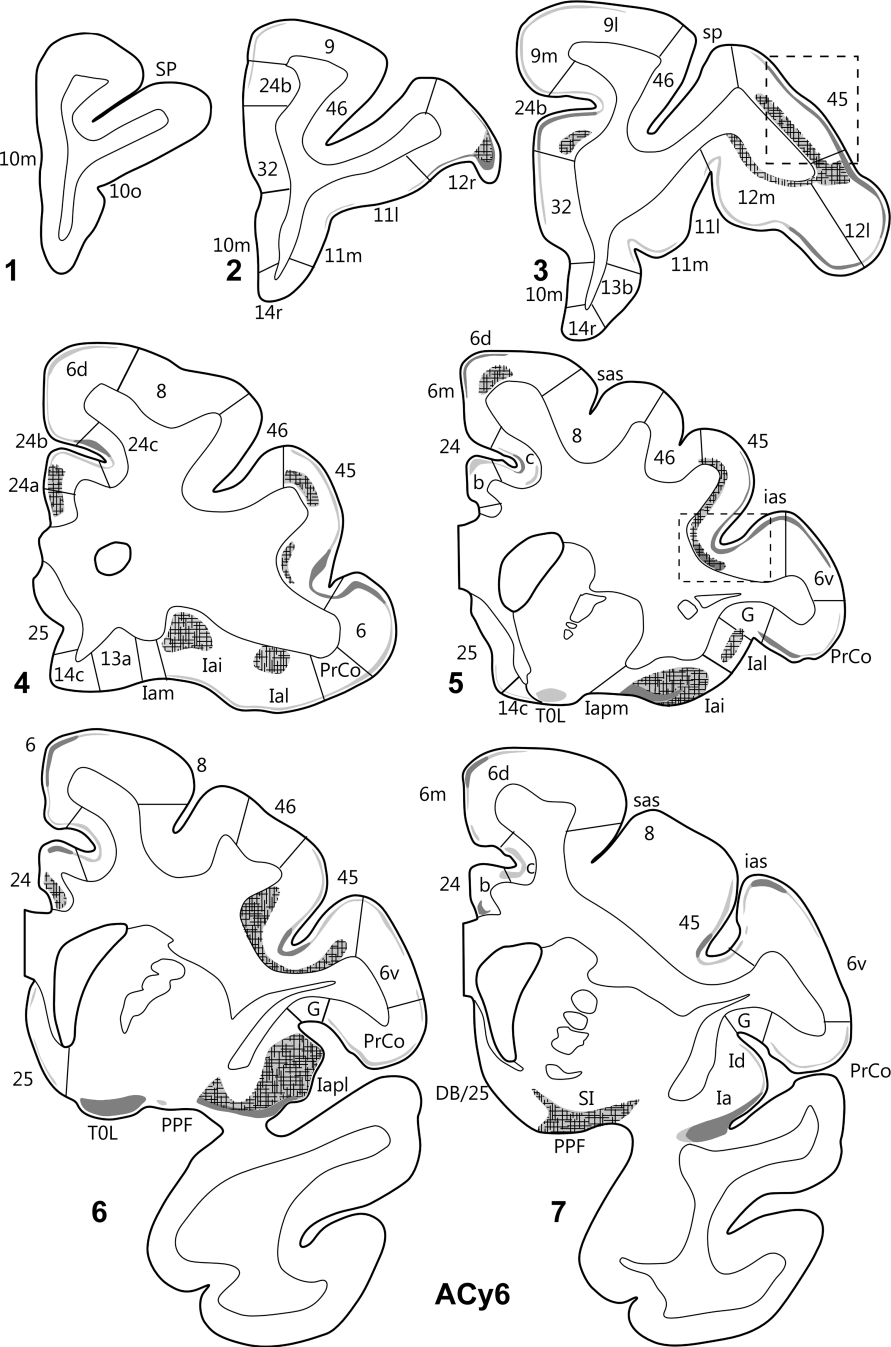
Projections from the intermediate and magnocellular parts of the basal amygdala nucleus. Series of drawings of coronal sections from case ACy6 going from anterior (#1) to posterior (#7). The numbers (letters in the case of the insula) correspond to different prefrontal areas. Terminal label is shown in gray, with darker gray representing denser label. The cross-hatching marks those areas with both fiber and terminal labeling. SI, substantia innominata; other abbreviations are as in Figure 3.

Further information came from case ACy6 where the amino acid injections were again centered in the intermediate and magnocellular basal nucleus but appeared to involve adjacent parts of the lateral and accessory basal nuclei. Because the injections were confined to one hemisphere (unlike ACy21), it was possible to look for any crossed projections to the prefrontal cortex. In fact, no crossed terminations were observed. The overall distribution and lamina pattern of the label in ACy6 (Fig. 7) closely matched those described for ACy21, although the label was slightly less widespread as there was no evidence of a projection to area 46 in case ACy21 (Tables 1–3). The input to area 25 appeared restricted to layer I in the ventral part of posterior area 25, despite the many labeled fibers passing deep (i.e., lateral) to area 25. While the label in areas 45 (Figs 5*B*, 6*C*) and 12 appeared denser in ACy6 than ACy21, the opposite was the case for the label in area 24 and PrCo.

Two cases (ACy10 and ACy13) had injections centered in the parvicellular division of the basal nucleus (Fig. 1). In case ACy10, the injection extended dorsally to the border with the intermediate division of the basal nucleus and ventrally to just reach the deepest layer of the immediately adjacent entorhinal cortex (Figs 1 and 2). Only restricted label was present on the orbital surface. Anterior to the genu, light label was present in the deep layers (V and VI) of area 13b, which continued behind the genu. Labeled fibers and probable termination were also found across the deep layers (V and VI) of the agranular insula, becoming increasing dense in more posterior sections. The medial surface contained most of the transported label in case ACy10 (areas 24b and 32), which began anterior of the genu. The most prominent label was in area 24b along the lower bank of the cingulate sulcus, where label was present in all layers except layer I, whereas the label in layer II was densest. This area 24b label gradually became lighter approaching the genu of the corpus callosum, where it ceased. Label was also present in the adjacent area 32 which, in contrast to area 24b, became denser closer to the genu. The label in area 32 was densest in layers II, V, and VI, with light label in layer III. Immediately behind the genu, there were labeled fibers deep to area 32, as well as a terminal label in area 14c (layers V and VI). The deep label in area 13b continued posterior of the genu. More caudal in case ACy10 the label in 14c became increasingly dense, so that it was found across all layers except layer I, but was most dense in V and VI. Dorsal to area 14c many fibers were visible just deep to area 25, but there was no definite evidence of termination in this area. Just above the dorsal limit of area 25 labeled fibers could be seen passing through the tenia tecta. Label was not observed in the lateral or dorsal prefrontal cortex (in case ACy13, the injection just reached uncal CA1 and so is not described, although the distribution of label corresponds to that in case ACy10.)

**Figure 8.**
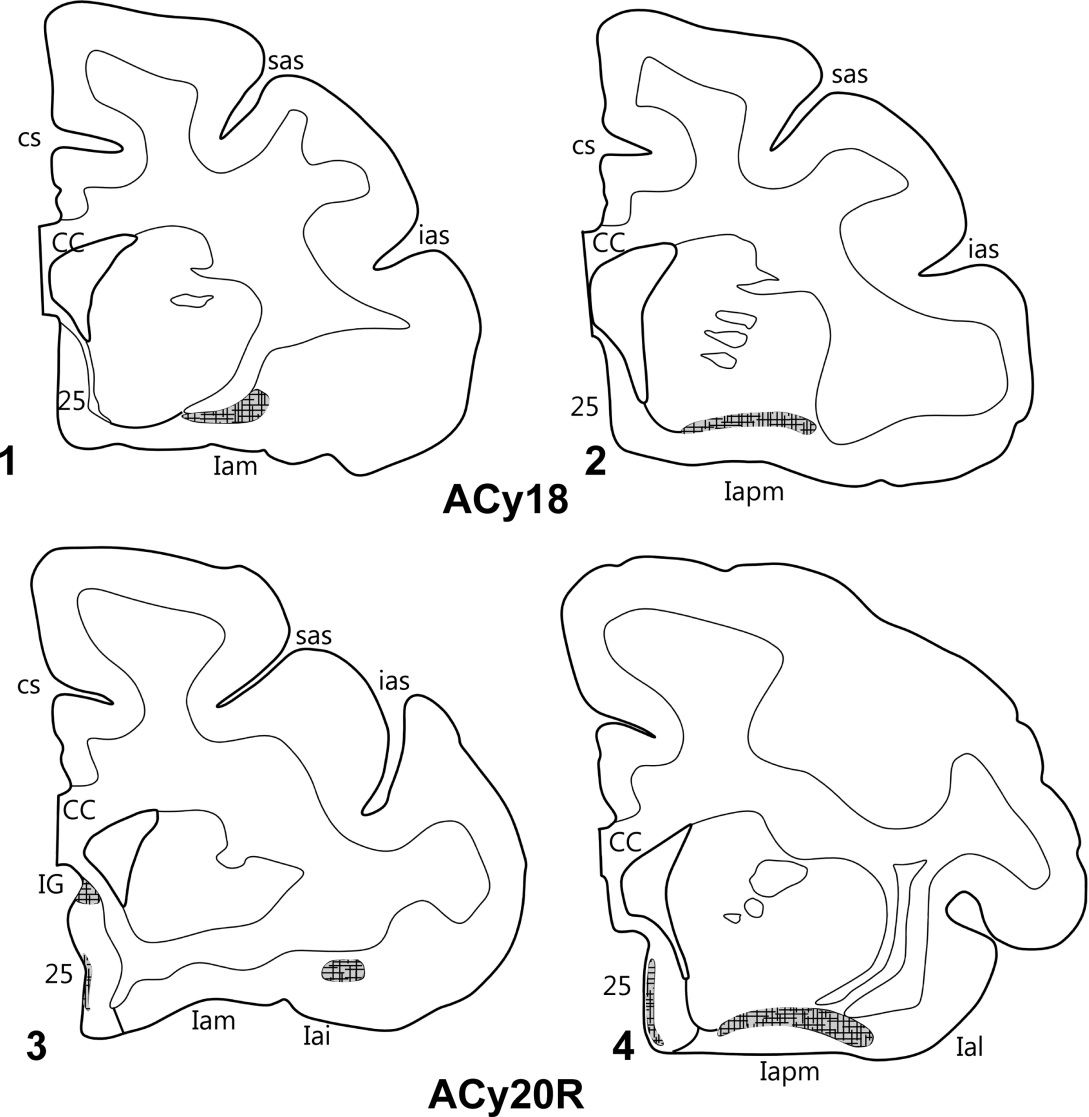
Projections from the accessory basal amygdala nucleus. The coronal sections from 2 cases (ACy18, upper: ACy20R, lower) show the restricted areas of label. In all areas, the label consisted of fibers with apparent terminal labeling and so is depicted with cross hatching. The numbers (letters in the case of the insula) correspond to different prefrontal areas. CC, corpus callosum; cs, cingulate sulcus; IG, induseum griseum; other abbreviations are as in Figure 3.

In 3 hemispheres, an injection (Fig. 2) was placed in the accessory basal nucleus (cases ACy20L, ACy20R and ACy18). Again, in none of these cases was there label in the lateral prefrontal cortex, nor was there any label anterior to the genu of the corpus callosum. Below the genu, a small area of labeled fibers was present around the induseum griseum in ACy20R (Fig. 8), but the only evidence of termination was in the posterior subcallosal gyrus and in the posterior agranular insula area. Labeled fibers were present just deep to posterior area 25 in all 3 hemispheres, along with evidence of light terminal label in deep layer I of area 25 (which sometimes reached superficial layer II). At the very posterior limit of area 25, this subcallosal label sometimes spread across all layers, probably reflecting labeled fibers. All 3 hemispheres with accessory basal injections also had a light band of label, much of it fibers, across the deeper parts of Iapm, Iai, and Ial (Fig. 8). Of the 3 injection cases, the insula label was lightest in ACy20L.

Two cases had injections essentially confined to the lateral nucleus of the amygdala (ACy16 and ACy22R). In the case with the larger injection (centered in the ventral part of the lateral nucleus—ACy16), labeled fibers were evident in the white matter just deep to subcallosal area 25. Labeled fibers cut across all cell layers of posterior area 25, although any termination appeared very light. There was a limited patch of label in layer I of the rostral agranular insula area Iam. No other frontal label was visible. The pattern of label in ACy22R (injection centered in dorsal part of lateral nucleus) was even more restricted. The only label was present in the molecular layer of the frontal operculum, associated with the prepiriform cortex and the olfactory tubercle (TOL 1—see Turner et al. 1978). Some of this layer I label appeared to reflect axons rather than termination.

In one case, ACy22L, the injection largely involved the medial nucleus. Label was present in the frontal operculum, but this was essentially restricted to layer I of the olfactory tubercle and layer I of the prepiriform cortex. A few labeled fibers were also present in layer I of posterior area 25. In the final case (ACy17), the injection largely involved the central nucleus but reached the dorsomedial border of the basal nucleus and the dorsal accessory basal nucleus. In this case (ACy17), there was no apparent termination rostral to the genu of the corpus callosum. At the level of the genu, light label appeared in medial area 13l, 13m, and 13a, which continued medially so that label was also present in area 25 and at the border between area 14r and 14c. In all cases, the label was in layer I. Moving a little more posterior, the label in areas 14c and 25 became much more pronounced (the area 25 label sometimes being most evident in the ventral half of the area 25). At these more posterior levels, layer I label was also present across all portions of the agranular insula. Thus, this case stood out for the way that the terminal label was essentially confined to layer I.

### Hippocampal Projections

**Figure 9.**
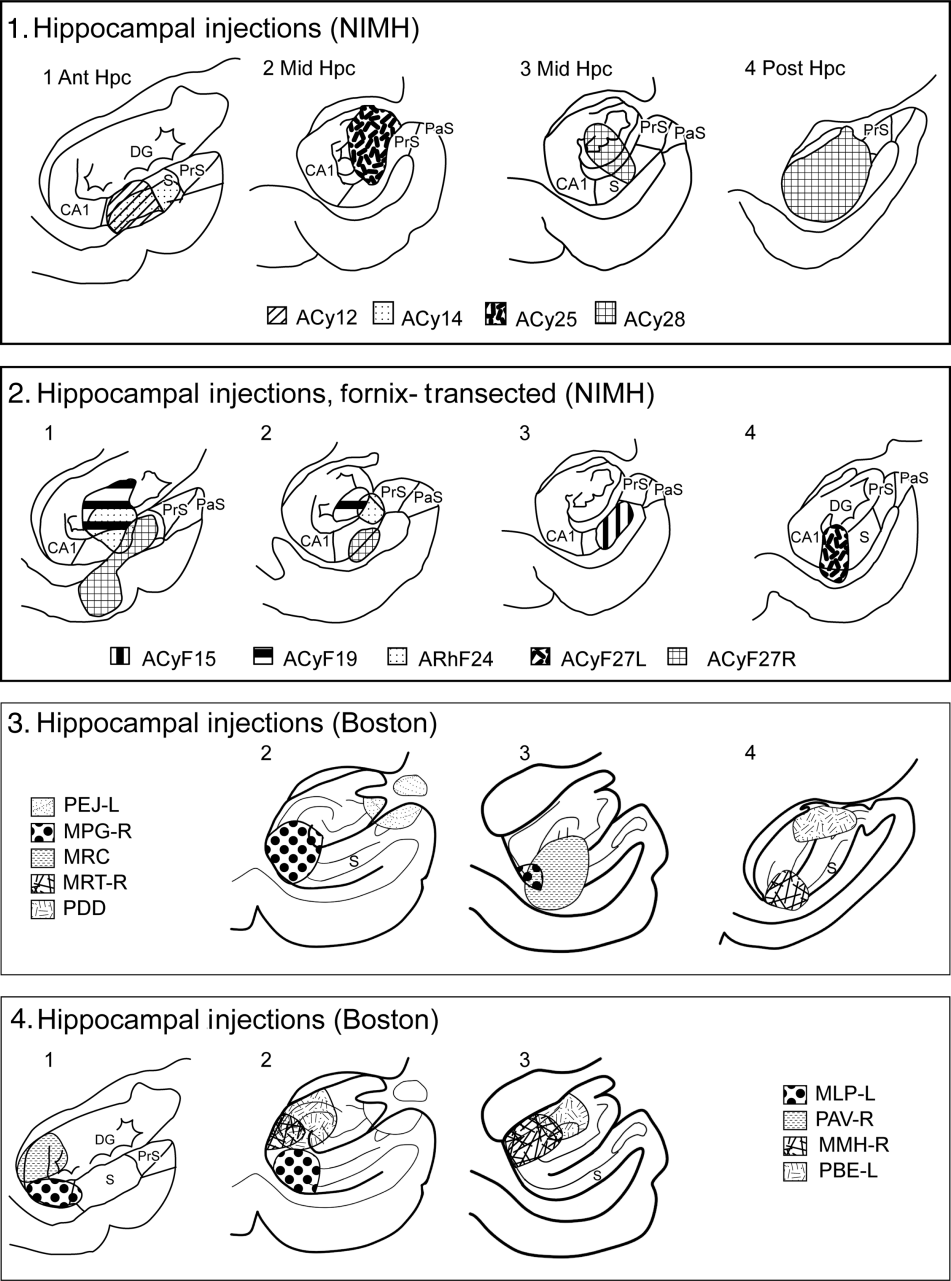
Extent of the core of each amino acid injection in the hippocampal formation drawn onto standard coronal sections. The cases are divided between the 2 cohorts (from NIMH or Boston University). The injection sites in the normal animals from NIMH are depicted in the upper row, whereas the second row shows those NIMH cases where the fornix was transected prior to injection. The lower 2 rows show the cases from Boston University. CA1, hippocampal field CA1; DG, dentate gyrus; Hpc, hippocampus; PaS, parasubiculum; PrS, presubiculum; S, subiculum.

Figure 9 depicts the placement and extent of the injections of tritiated amino acids involving the hippocampus. The initial descriptions of prefrontal label are based on the results from 5 cases. These cases were selected as each had frontal label in multiple sites, and they respectively involved efferents from the anterior (ACy12 and ACy14), mid (MLP-L and MRC), and posterior (ACy28) levels of the hippocampus. It was immediately apparent that the hippocampal projections were far more restricted and typically far lighter than those from the amygdala.

**Figure 10.**
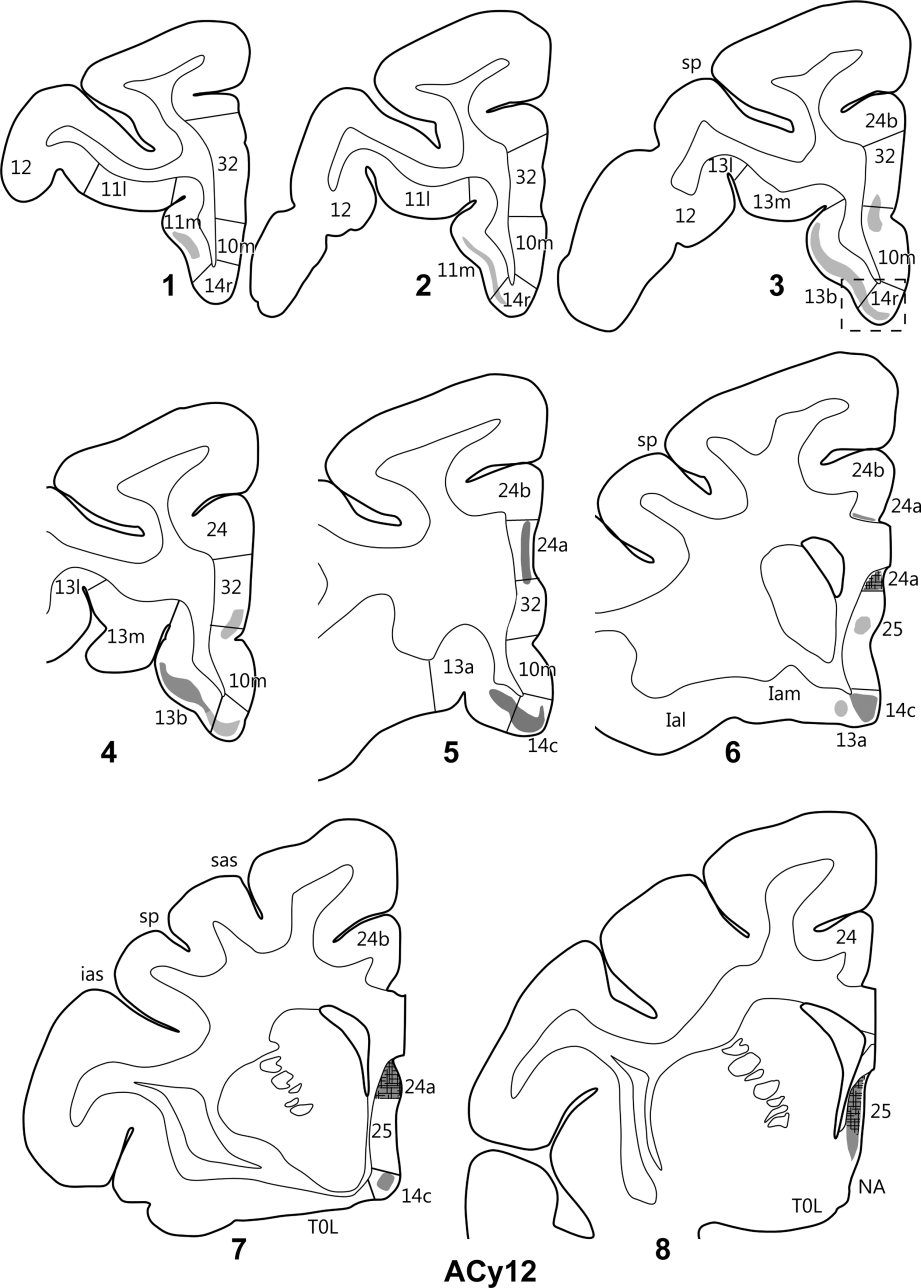
Projections from the anterior subiculum of the hippocampal formation in case ACy12. The series of drawings of coronal sections go from rostral (#1) to caudal (#8). The numbers (letters in the case of the insula) correspond to prefrontal areas. Terminal label is shown in gray, with darker gray representing denser label. The cross-hatching marks those areas with both fiber and terminal labeling. NA, nucleus accumbens; other abbreviations are as in Figure 3.

**Figure 11.**
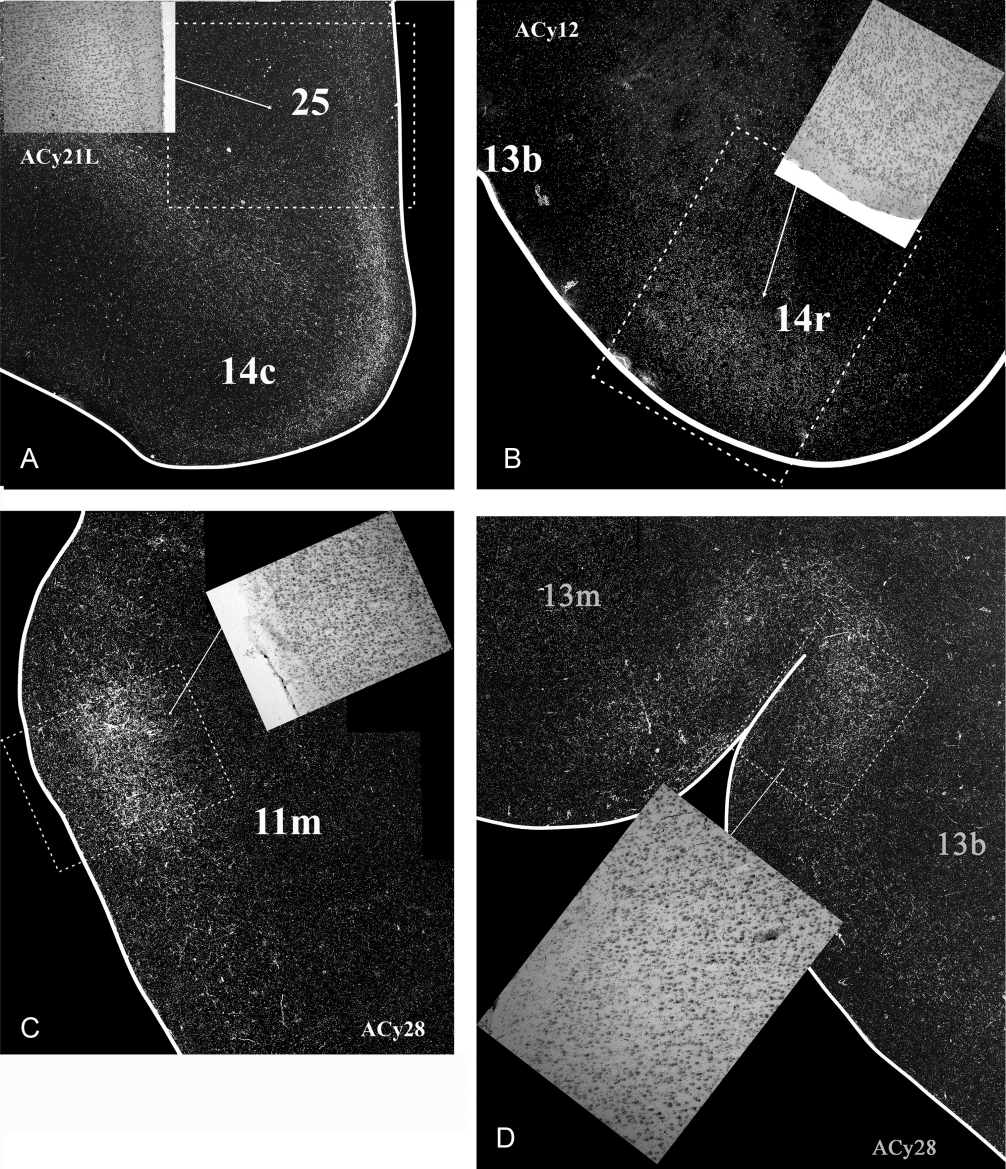
Darkfield images of autoradiographic label in 2 cases with amino acid injections in the hippocampus (ACy12, anterior subiculum; ACy28, posterior hippocampus). For purposes of comparison, a case with an amygdala injection (ACy21L) is shown in box A. All hippocampal projections (*B,C,D*) are on the orbital surface. The brightfield images correspond to the subregions marked by boxes with dashed lines. The entire area of each darkfield image is indicated in Figure 3 (box A), Figure 10 (box B), and Figure 13 (boxes C and D).

An injection centered in the anterior prosubiculum/subiculum (case ACy12) that reached the distal CA1 border, that is, included all of the prosubiculum (Figs 2,9)-contained label in the medial orbital prefrontal cortex, which was typically denser in its more posterior regions. Starting in the rostral prefrontal cortex, light terminal label was present in layer III of area 11m (Fig. 10), which continued posteriorly in this layer into area 13b. The label in 13b reached into the lateral banks of the medial orbital sulcus and so just included the most medial parts of area 13m. The area 13 label became appreciably denser going posteriorly and, at the same time, light label appeared at the transition area between 14r and 14c (Fig. 11*B*). The area 13 label continued posteriorly to include 13a, such that this label was continuous with that in area 14c. This label in 14c was denser than that in the rest of the orbital cortex. A consistent feature of the label in areas 11 and 13 was that it was diffusely scattered across layer III. The label in area 14 was again largely in layer III, but at the most posterior parts of area 14c, label was found across all levels, except for layer I. Some but not all of this deeper label in area 14c was from fibers. No definite terminal label was found in orbital insula areas.

The label in the medial prefrontal cortex in case ACy12 was predominantly found just anterior to the genu and below the genu of the corpus callosum (Fig. 10). The most anterior label was diffusely spread across layer III in those parts of area 32 next to the genu and immediately above the rostral sulcus. This light label was joined by label in 24a immediately in front of the genu, which continued posteriorly just above and below the corpus callosum. Below the callosum, label was also found in area 25 that was often more concentrated in the dorsal parts of the area. The label in area 25 increased going more posteriorly, so that the most posterior parts of area 25 were full of label that again became denser going dorsally within the area and which involved all cell layers, aside from layer I. Many labeled fibers were present immediately deep to area 25, with much of the label within area 25 itself also reflecting fibers of passage.

**Figure 12.**
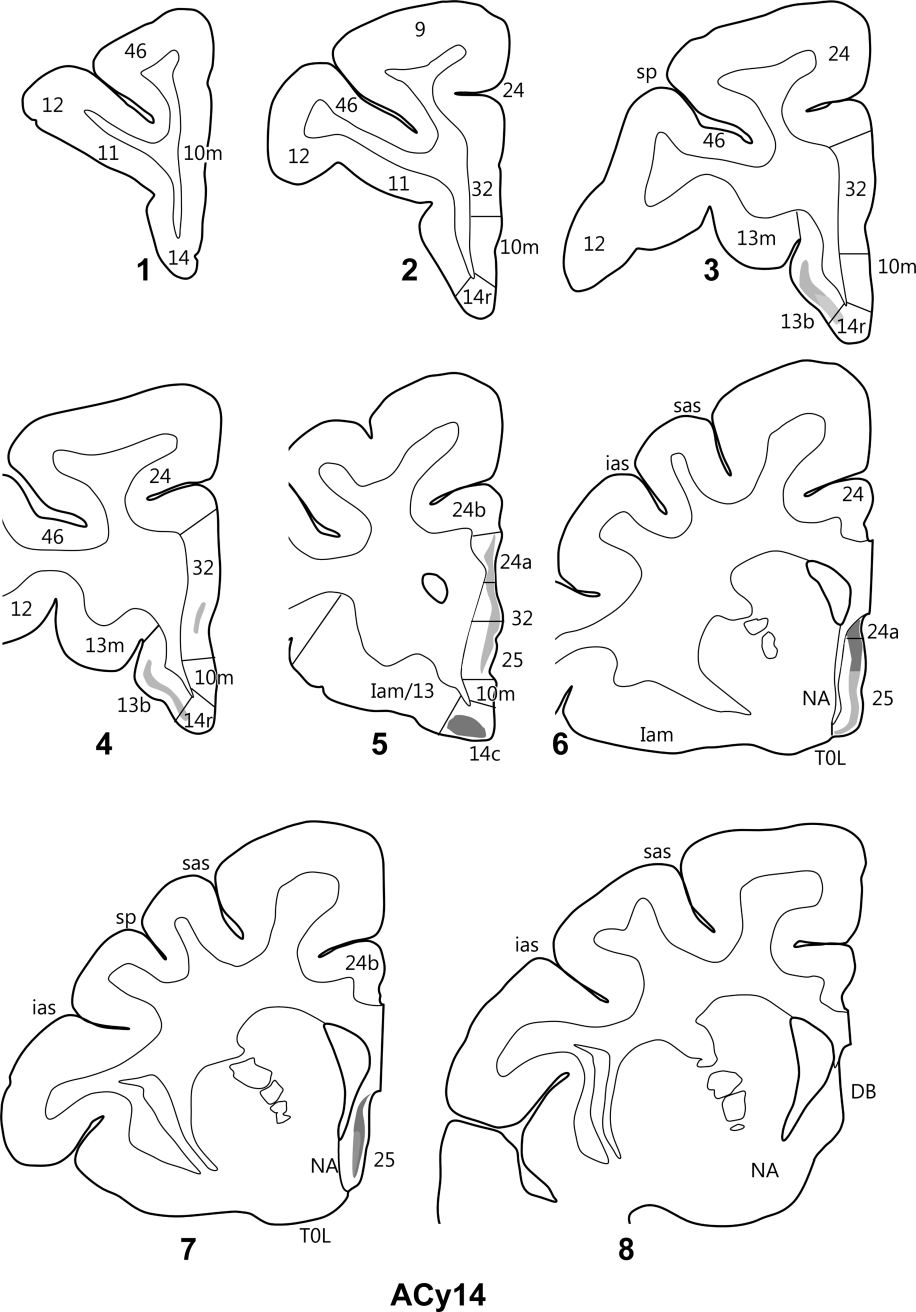
Projections from the anterior subiculum of the hippocampal formation in case ACy14. The series of drawings of coronal sections go from anterior (#1) to posterior (#8). The numbers (letters in the case of the insula) correspond to different prefrontal areas. Terminal label is shown in gray, with darker gray representing denser label. The cross-hatching marks those areas with both fiber and terminal labeling. NA, nucleus accumbens, DB, diagonal band; other abbreviations are as in Figure 3.

In a second case (ACy14), the injection was again placed in the anterior subiculum (Fig. 9) but was located a little more distal to CA1 than the previous case (ACy12). Consequently, the injection in ACy14 reached the border with the presubiculum but did not appear to involve any of CA1. In the orbital cortex, light diffuse label was seen in layer III of 13b, which extended medially to just reach into layer III of area 14r (Fig. 12). No label was seen in area 11. More caudal, light label was seen in layer III of 14c, with even lighter label in other layers. Very light label was also present in layer III of the adjacent area 13a. On the medial wall of the prefrontal cortex, light label was found in area 24a just in front of the genu in layer III (note layers II and III are virtually indistinguishable in areas 24a) and below that in layer III of areas 25 and 32 (Fig. 12). This subcallosal label became denser going posterior and dorsal within area 25. Transported label from the hippocampal injections filled the most caudal area 25, except for the molecular layer, and this label appeared to consist of both termination and fibers of passage. Again, there was no clear evidence of terminal label in the orbital insula.

Two cases (MLP-L and MRC) had more posterior injections in the subicular cortex that involved mid-AP levels of the hippocampus. The injection in case MLP-L was centered at the prosubiculum/CA1 border but extended into the subiculum (Fig. 9). It extended from anterior to mid-AP levels within the hippocampus. The overall pattern of label was similar to that in ACy12, but more restricted. Label was seen in that part of area 13m in the medial wall of the medial orbital sulcus, although the label continued into area 13b more posteriorly. The label in area 13 was in layer III. Behind the genu, there was a restricted patch of label in area 24a immediately above the callosum (layer III), whereas, below the callosum, the label extended into dorsal 25. Only at the posterior limits of area 25 did the label become more extensive, where it occupied the cellular layers of area 25. An area of light label (fibers and apparent termination) was also present in parts of area 14c. Here, light label was evident in all layers except I and II.

In case MRC, the injection was centered in distal CA1 and the immediately adjacent prosubiculum, reaching proximal subiculum, but was more caudally placed within mid-hippocampal levels than case MLP-L (Fig. 9). The prefrontal label in MRC was even more restricted than that in case MLP-L. In case MRC, a few labeled fibers were seen deep to area 32 in front of the genu, but there was no evidence of termination. In contrast, there was evidence of light termination in 13b (layer III) that also involved 13a and continued into adjacent area 14c. Some fibers were also labeled immediately deep to caudal area 25.

**Figure 13.**
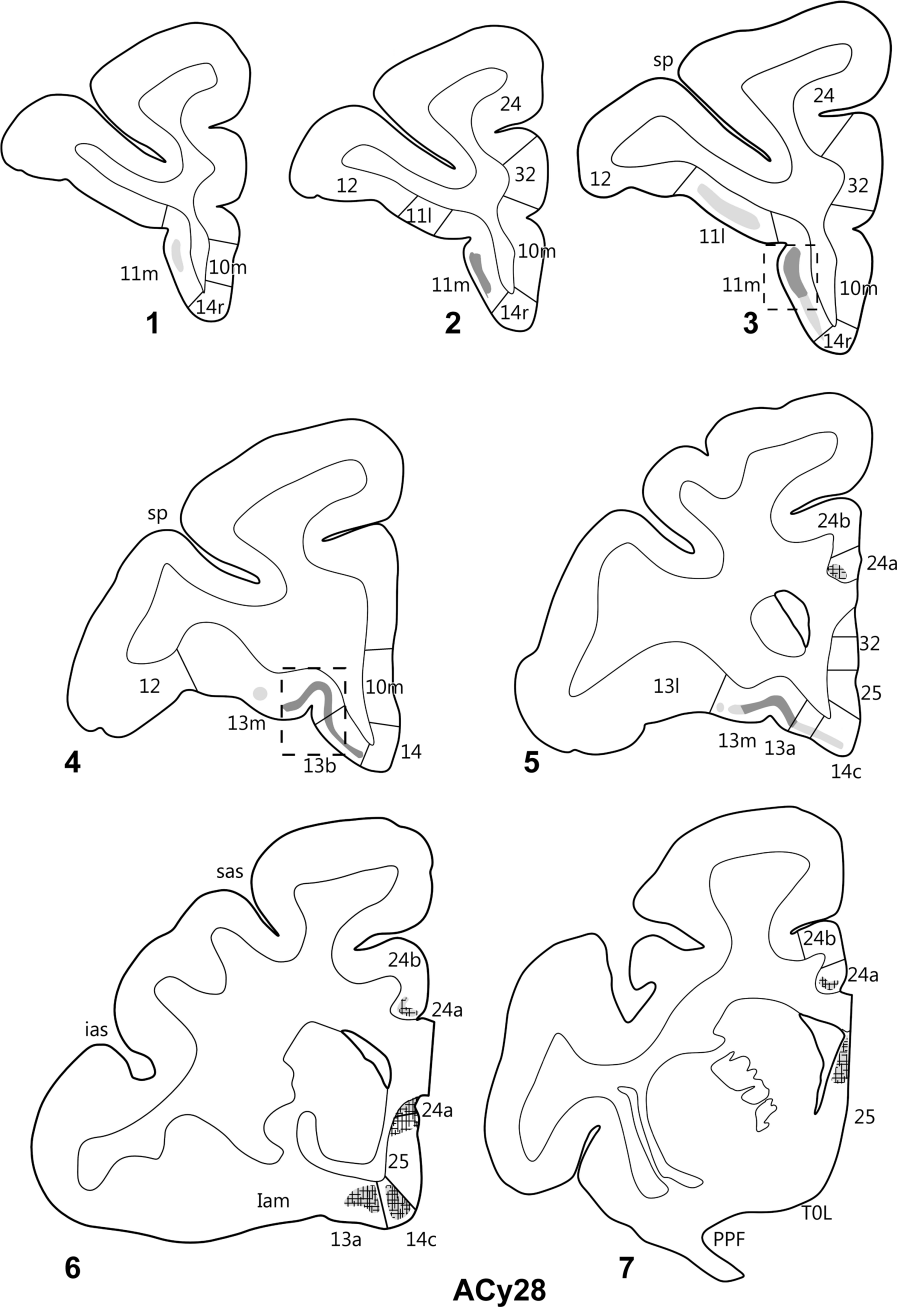
Projections from the posterior hippocampal formation in case ACy28. The series of drawings of coronal sections go from anterior (#1) to posterior (#7). The numbers (letters in the case of the insula) correspond to prefrontal areas. Terminal label is shown in gray, with darker gray representing denser label. The cross-hatching marks those areas with both fiber and terminal labeling. Abbreviations are as in Figure 3.

A large injection was placed in the posterior hippocampus in ACy28 (Figs 2,9). Unlike any other case, this injection involved almost all of the hippocampal fields, from the dentate gyrus to the subiculum as well as the most proximal presubiculum. Despite the injection being considerably larger than all previous cases (Fig. 9), the prefrontal label was no more dense or extensive than that seen after the 2 rostral injection cases (ACy12 and ACy14). Once again the cortical label was diffusely scattered across layer III (unless otherwise stated), becoming denser in the more posterior parts of the various regions (Fig. 13). The orbital label began in the middle of 11m (Fig. 11*C*) and continued posteriorly to fill layer III of area 13b, the medial part of area 13m (Fig. 11*D*), and area 13a. There was a sparse scattering of label in the posterior region of 14r (layer III), although this label became appreciably denser in area 14c. Area 14c was filled with a mixture of fibers and termination, with most label across layers III–VI. Much of the label in the deeper layers comprised fibers. On the medial cortex, many fibers skirted around the genu and ran ventrally just lateral to areas 32 and 25. Label was also present across all cellular layers of posterior 25, which appeared to be a combination of fibers and termination (Fig. 13). Immediately dorsal to the anterior corpus callosum, there was a light patch of label, which included fibers, in area 24a. This label was scattered across layers III and V, along with labeled fibers immediately dorsal to the induseum griseum. Despite adjacent passing fibers, no clear-cut terminal label was found in orbital insula areas.

The presubiculum was injected in 2 cases at different anterior–posterior levels, though in both cases, the injection did not reach the deepest cellular layers. In case PEJ-L, an injection at the mid-AP level of the hippocampus filled much of the presubiculum, along with adjacent parts of the dentate gyrus (Fig. 9). No prefrontal label was found in this case (though sections were not available at the frontal pole). An injection in case PDD-L (Fig. 9) filled the posterior presubiculum, along with parts of the dentate gyrus, but again, no prefrontal label was observed.

Other information came from those cases where there was no discernible prefrontal label. As might be expected, a large injection into fields CA3/CA2 at mid-hippocampal levels (case PBE-L) led to no prefrontal label (see Barbas and Blatt 1995). More surprising, however, are those cases where the injection involved the CA1 field yet again no prefrontal label was visible. In case PAV-R, an injection centered in the anterior part of proximal CA1 (Fig. 9) resulted in no apparent prefrontal label, aside from some labeled fibers deep to area 25, some of which terminated in nucleus accumbens, and some label in the induseum griseum. A lack of prefrontal label was also found for 2 cases (MPG-R and MMH-R) with injections in CA1 at the mid-anterior–posterior hippocampal level (Fig. 9). In case MMH-R, the injection was in proximal CA1 (at the CA2 border), whereas in MMH-R, the injection was placed centrally within CA1. Likewise, a further case (MRT-R) with injections in the posterior hippocampus that involved CA1 and the adjacent prosubiculum also failed to show prefrontal label.

#### Fornix Transection Cases

None of the cases with fornix transection and injections involving the subiculum, prosubiculum, or CA fields (Fig. 9) contained any prefrontal cortex label (ACyF15, ACyF19, ARhF24, and ACyF27L), although comparable injections in intact monkeys (Fig. 2) resulted in appreciable prefrontal label.

## Discussion

The termination sites of the direct projections from the amygdala and hippocampus to the prefrontal cortex were compared in macaque monkey brains. Only ipsilateral projections were observed. In this regard, these prefrontal connections appear consistent with other ipsilateral cortical projections from the monkey hippocampus and amygdala, e.g., to parahippocampal cortical areas, temporal association cortex (amygdala), and retrosplenial cortex (hippocampus) (Amaral and Price 1984; Aggleton et al. 2012). The dominance of ipsilateral cortical connections is reinforced by the scarcity of interhemispheric connections in the primate brain that directly link the amygdala and hippocampus with their counterparts in the opposite hemisphere (Demeter et al. 1985). In contrast, some subcortical hippocampal and amygdala projections in the macaque brain have an evident-crossed component, for example, to the septum (hippocampus), mammillary bodies (hippocampus), and thalamus (amygdala and hippocampus) (Aggleton and Mishkin 1984; Demeter et al. 1985; Aggleton et al. 1986, 1987, 2005; Russchen et al. 1987). Of these connections, the crossed hippocampal projections to the anterior thalamic nuclei are especially plentiful (Aggleton et al. 1986), seemingly giving these connection a special status.

**Figure 14.**
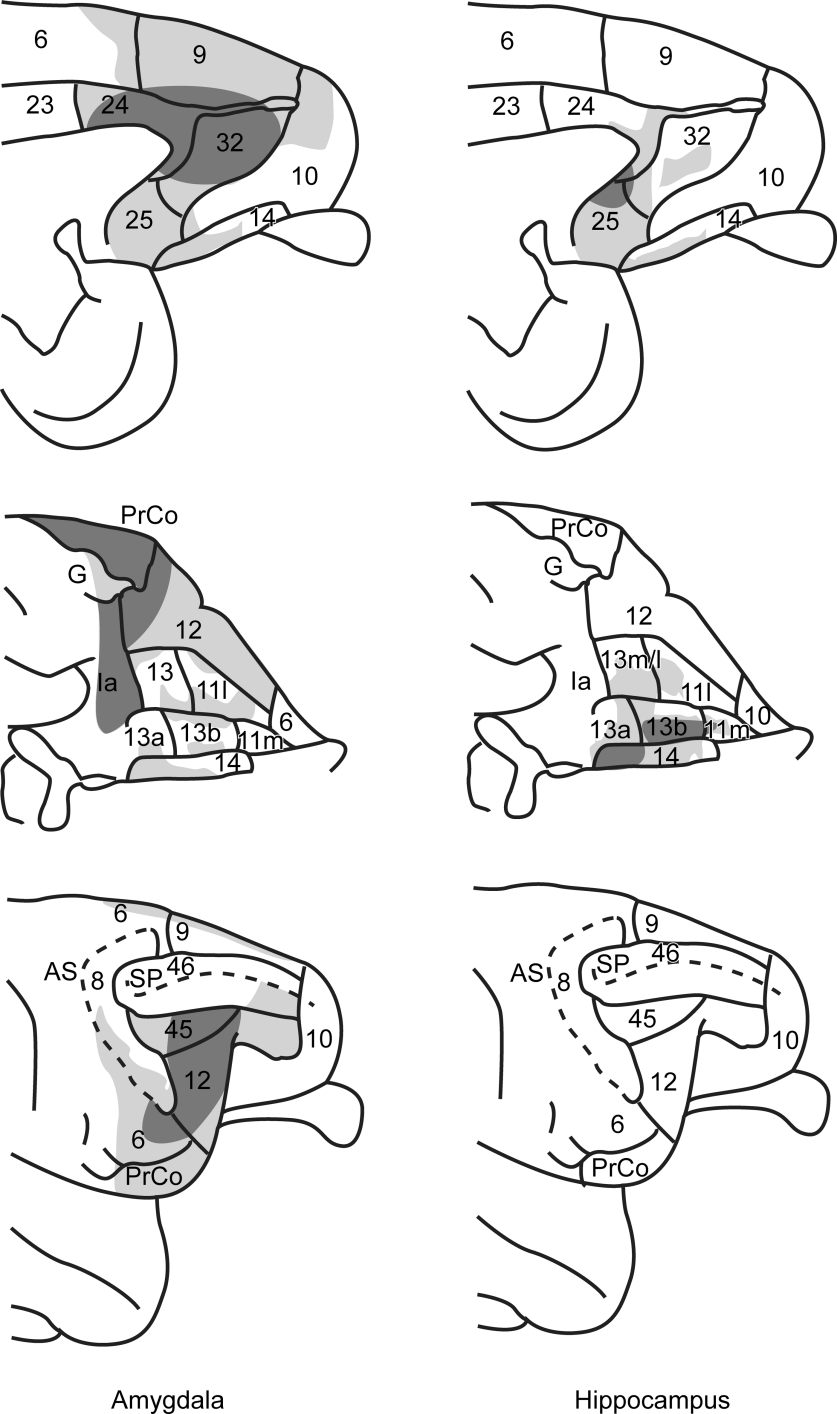
Summary figure depicting the termination sites of the amygdala (left) and hippocampal formation (right) projections to the medial (top), orbital (mid), and lateral (bottom) surfaces of the prefrontal cortex. The area boundaries and nomenclature come from Carmichael and Price (1994). The darker gray shading corresponds to the more dense terminal label. Decisions about the density of label were made by 2 independent observers. The figure shows both the extent of the projections and the limited numbers of areas with joint inputs from both structures. AS, arcuate sulcus; other abbreviations are as in Figure 3.

The amygdala gives rise to widespread projections to medial, orbital, and lateral parts of prefrontal cortex, which appeared more extensive than described in previous autoradiographic studies (Porrino et al. 1981; Amaral and Price 1984) and were often more comparable with those detailed with BDA (Ghashgaei et al. 2007). These amygdala projections principally arose from the intermediate and magnocellular portions of the basal nucleus, that is, the lateral basal nucleus (Crosby and Humphrey 1941). In contrast, the hippocampal projections, which were strongly associated with injections involving the subiculum, terminated in a far more restricted set of medial and orbital prefrontal sites, with no evidence of any lateral prefrontal projections. All of these hippocampal projections to prefrontal cortex appeared wholly dependent on the fornix. A striking feature was the limited overlap between amygdala and hippocampal termination sites (Fig. 14). Even when both structures projected to the same area, their projections typically occupied different lamina (Fig. 11).

This discussion first considers the hippocampal projections as their termination sites remain poorly understood. The hippocampal projections to orbital and medial areas 11, 13, 14r, and 32 terminated in layer III, with the inputs to areas 14c and 24a primarily targeting layer III, but also including other layers. While the hippocampal inputs to area 25 again included layer III, the projections to this area more evenly involved other layers. However, area 25 also contained an unusual concentration of fibers of passage, especially in its most caudal portions, sometimes making it difficult to specify the lamina of terminal label. Other findings included the discovery that the hippocampal inputs to cingulate area 24 are confined within area 24a. Hippocampal projections to area 24 had been demonstrated with retrograde tracers (Carmichael and Price 1995; Insausti and Munoz 2001), although it was not possible to specify the limits of the termination field with this technique. The use of anterograde tracers also provided a new perspective on conflicting reports of hippocampal projections to orbital parts of the insula cortex. Such projections have been reported in some (Barbas and Blatt 1995), but not all (Carmichael and Price 1995), retrograde tracer studies. The present study found that although numerous hippocampal fibers lie just deep to this cortical region, no definite terminal label could be found in the insula cortex. The implication is that at least some of the retrograde label reported in the hippocampus reflects uptake from this immediately adjacent white matter.

By combining anterograde tracers with surgical section of the fornix, it was possible to demonstrate that the hippocampal projections to the macaque prefrontal cortex rely exclusively on the fornix. Previous degeneration studies in squirrel monkeys had shown prefrontal inputs from the hippocampal formation that involve the fornix (Poletti and Cresswell 1977), although that procedure could not determine whether there were additional, nonfornical routes. The present conclusion does, however, assume that cutting the fornix spares the transport of amino acids by other (nonfornical) routes from the hippocampus. In fact, fornix transection in monkeys does not appear to cause hippocampal cell loss (Daitz and Powell 1954), while examination of the cases used in the present study showed that hippocampal cells remain capable of transporting amino acids long after the fornix lesions. Examples include hippocampal projections to sites such as the amygdala and retrosplenial cortex (Aggleton 1986; Aggleton et al. 2012). While fornix lesions in rats can produce neuroplastic responses in the hippocampus, which include sprouting (e.g., Booze and Davis 1987; Fass and Stein 1987), the lack of any nonfornical pathways to prefrontal cortex in the present study would indicate that sprouting is not a concern. The conclusion is, therefore, that the fornix provides the route for seemingly all direct hippocampal (subicular/CA1) projections to the macaque prefrontal cortex. The fornix also provides the route for almost all hippocampal projections to the anterior thalamus, mammillary bodies, and ventral striatum (Aggleton et al. 1986, 2005; Friedman et al. 2002). This array of connections helps to explain the disruptive effects of fornix damage upon episodic memory (Gaffan et al. 1991; McMackin et al. 1995; Tsivilis et al. 2008).

Retrograde tracing studies have revealed an anterior–posterior gradient in the hippocampal projections to the prefrontal cortex, with more numerous inputs arising from the anterior hippocampus (Barbas and Blatt 1995; Carmichael and Price 1995). Evidence for a similar pattern occurred in the present study as relatively more prefrontal projections were found in those cases with anterior hippocampal injections. In contrast, some posterior injections revealed very few, or even no, prefrontal projections. A similar anterior–posterior gradient is seen in the source of some other hippocampal efferents, including the direct projections to the perirhinal cortex, amygdala, and nucleus accumbens (Aggleton 1986, 2012; Saunders and Rosene 1988; Friedman et al. 2002). The hippocampal projections to these 3 sites share an additional property with the efferents to the prefrontal cortex, namely that inputs to all 4 sites predominantly arise from the distal CA1 and adjacent proximal subiculum (Barbas and Blatt 1995; Carmichael and Price 1995; Aggleton 2012). In contrast, other hippocampal projections, for example, to the dorsal retrosplenial cortex, parahippocampal cortices (TH and TF), and mammillary bodies, predominantly arise from the posterior hippocampus (Aggleton 2012). These contrasting properties provide anatomical features that presumably underpin functional divisions along the anterior–posterior axis of the hippocampus (Columbo et al. 1998; Fanselow and Dong 2010; Strange et al. 2014), which may include more coarse, global representations in the anterior hippocampus that contrast with higher-resolution, local representations in the posterior hippocampus (Poppenk et al. 2013).

Consistent with previous studies, there was no evidence that the dentate gyrus, CA3, or CA2 provide prefrontal inputs (see also Rosene and Van Hoesen 1977; Barbas and Blatt 1995; Carmichael and Price 1995). The present injections into the presubiculum also failed to reveal frontal projections, supporting the previous finding that the prefrontal projections from this area almost exclusively arise from the very posterior limit of the presubiculum, that is, close to the transition with retrosplenial cortex (Goldman-Rakic et al. 1984; Barbas and Blatt 1995). Furthermore, although some hippocampal formation projections to the lateral prefrontal cortex have previously been described (Goldman-Rakic et al. 1984; Barbas and Blatt 1995), these efferents again principally arise from the most posterior part of the presubiculum. A part of this region, which is posterior to the tracer injections in the present study, is contentious as other studies regard it as ventral retrosplenial cortex (e.g., Kobayashi and Amaral 2000), which has more widespread prefrontal connections (Morris et al. 1999; Kobayashi and Amaral 2007). More surprising, therefore, was the apparent lack of projections from the main body of CA1 to prefrontal cortex, given that some previous macaque studies using retrograde tracers have described how both the distal CA1 field and the adjacent proximal subiculum (prosubiculum) are the principal sources of the medial and orbital prefrontal projections (Morecraft et al. 1992; Barbas and Blatt 1995). Frontal projections from CA1 have also been described in other species, including marmosets (Roberts et al. 2007) and rats (Jay and Witter 1991; Cenquizca and Swanson 2007). There are several explanations for this apparent discrepancy in the findings for macaque brains.

The first explanation is that any CA1 projections predominantly arise from the most anterior hippocampus (Barbas and Blatt 1995; Carmichael and Price 1995; Insausti and Munoz 2001), consequently posterior CA1 injections will show few if any prefrontal projections (e.g., cases MPG-R and MMH-R). A second reason is that the CA1 efferents arise from the most distal part of the subfield, that is, at the prosubiculum border. Clearly, the precise placement of the CA1 border will alter the apparent number of projections arising from this transition area. In the present study, the prosubiculum is allied with the subiculum, rather than CA1 (see Ding 2013). Furthermore, if the CA1 border is placed at right angles to the alveus, that is, in direct alignment with the apices of the pyramidal cells (e.g., Barbas and Blatt 1995), then distal CA1 is likely to include deep cells that may, in fact, belong to the proximal prosubiculum, a point highlighted by Carmichael and Price (1995). This demarcation problem arises because the CA1: Prosubiculum border has a sloping profile (e.g., Amaral et al. 1984; Carmichael and Price 1995; Insausti and Munoz 2001; Saleem and Logothetis 2007; Paxinos et al. 2009; Ding 2013), such that the deeper cellular layers of the proximal prosubiculum sit under the superficial parts of distal CA1. This sloping border, which was adopted in the present study, appreciably decreases the CA1 contribution while increasing the prosubiculum contribution to prefrontal projections, as the cortical inputs arise from the underlying cell layer (Carmichael and Price 1995). As a result, our findings most closely match those reports using retrograde tracers that emphasize how the hippocampal–prefrontal inputs arise from the prosubiculum and subiculum (Rosene and Van Hoesen 1977; Carmichael and Price 1995), with relatively few inputs from CA1.

The relative lack of direct hippocampal inputs to much of the prefrontal cortex raises the issue of whether indirect routes principally fulfill this function. Potential indirect routes are via parahippocampal cortical areas (Goldman-Rakic et al. 1984; Lavenex et al. 2002; Kondo et al. 2005; Munoz and Insausti 2005), the retrosplenial cortex (Morris et al. 1999; Kobayashi and Amaral 2007; Aggleton et al. 2012), the anterior and midline thalamic nuclei (Kievet and Kuypers 1977; Aggleton et al. 1986; Hsu and Price 2007), and the amygdala (Aggleton 1986; Saunders et al. 1988). Of these routes, those via the parahippocampal region and thalamus will primarily target medial and orbital frontal sites (Lavenex et al. 2002; Hsu and Price 2007), so providing overlap with the direct subicular efferents. For these reasons, the subicular projections to the retrosplenial cortex have added significance as they offer the hippocampus indirect routes to lateral prefrontal regions, areas that receive few, if any, direct hippocampal inputs (Morris et al. 1999; Kobayashi and Amaral 2007; Aggleton et al. 2012). These connections of the retrosplenial cortex, forming a way station between the hippocampus and prefrontal cortex, presumably contribute to the importance of the retrosplenial cortex for learning and memory (Vann et al. 2009; see also Prasad and Chudasama 2013).

The amygdala connections were very different from those of the hippocampus, with widespread, often dense, projections to prefrontal cortex (Fig. 14). These projections overwhelmingly arose from the basal nucleus, in particular its intermediate subfield. The same principal source is identified in retrograde tracer studies of macaque monkeys, which also show lighter projections arising from the accessory basal nucleus, along with limited inputs from the lateral nucleus to frontal insula areas (Jacobson and Trojanowski 1975; Porrino et al. 1981; Carmichael and Price 1995; Ghashgaei and Barbas 2002). Consistent with retrograde tracer studies, a striking contrast was seen in the present study between the lateral amygdala nucleus, with very limited prefrontal projections, and the immediately adjacent parts of the basal nucleus, with extensive prefrontal projections, despite the fact that both amygdala nuclei receive dense sensory inputs from temporal cortex (Herzog and Van Hoesen 1976; Aggleton et al. 1980; Amaral et al. 1992). Such findings highlight the likely functional importance of the numerous intra-amygdala connections from the lateral to the basal amygdala nuclei (Aggleton 1985; Amaral et al. 1992) if sensory information reaching the lateral nucleus is to influence large parts of prefrontal cortex.

The overall pattern of amygdala projections was in close agreement with a previous study using the same methodology and species (*Macaca fascicularis*) (Amaral and Price 1984). Indeed, every area reported by Amaral and Price (1984) to receive an amygdala input also contained label in the present study. In both studies, relatively dense projections from the basal nucleus were concentrated throughout areas 24, 25, and 32 on the medial surface and along areas 12 and 14 on the orbital surface. In the present study, particularly dense label was found within areas 24a and 24b of the anterior cingulate cortex, along with areas 12m, 12l, 45, PrCo, Ial, and Iapm. The study by Amaral and Price (1984) also reported light restricted amygdala projections to areas 6, 10, 13a, 45, and 46. Not only were these same projections observed in the present study but injections centered in the intermediate basal nucleus also revealed additional terminal label in parts of areas 6d, 9m, and rostral area 45.

More recently, Ghashgaei et al. (2007) reported the extent of prefrontal projections from the amygdala in rhesus monkeys (*Macacca mulatta*), giving particular emphasis to the patterns of lamina terminations. That study, which described the anterograde transport of BDA from 4 injection cases, differed in a number of key respects from the present experiments. The individual BDA injections were considerably more extensive, each involving multiple nuclei (Ghashgaei et al. 2007). While this feature helped to reveal the full extent of amygdala inputs across prefrontal cortex, it also made it more difficult to attribute specific projections to particular nuclei. In fact, the overall distribution of label described in the present experiment is remarkably similar to that reported by Ghashgaei et al. (2007), with the dorsolateral surface of the most rostral prefrontal cortex (adjacent parts of areas 9 and 10) in both studies being one of the only areas to receive few, if any, amygdala inputs. One difference with the report by Ghashgaei et al. (2007) is that the present study considered additional subregions within areas 11, 12, 13, 24, and insula cortex, to give a more fine-grained description of area termination.

For many areas, the projections from the intermediate and magnocellular basal nucleus preferentially terminated in layer II along with the deep (i.e., immediately adjacent) part of layer I (Tables 1–3 and Figs 5 and 6). Such sites included areas 45, 46, 12m, 12o, 12l, 13b, 13m, 6d, and 6v (Figs 5,6). This same pattern of termination in deep layer I and layer II is also seen in the widespread projections from the amygdala across much of the temporal cortex (Amaral and Price 1984). In the medial wall and some other prefrontal areas, there was additional label in that part of layer III immediately adjacent to layer II. This second pattern was seen in areas 24a, b, c, 32, PrCo, and 45, as well as the agranular insula cortex (Tables 1–3) (it should be noted that layer II is indistinct in some of these areas.) A third pattern was associated with those sites receiving especially dense projections, as these sites often contained additional label in layer VI, and sometimes layer V (Tables 1–3). This deeper label was especially evident in areas 32, 24b, 12, and 45, along with parts of area 6. Finally, broad columns of label were occasionally apparent across all layers in some areas (e.g., Figs 4(5), 5*B*, and 6*A*,*C*; see also Ghashgaei et al. 2007).

These findings for lamina termination in prefrontal areas agree with, and extend, the descriptions of Amaral and Price (1984). In doing so, they show strong similarities with Ghashgaei et al. (2007) who used BDA to determine lamina terminations. That study, which used quantitative methods, emphasized more strongly the density of the some of the inputs to the deep cortical layers (both V and VI) than that reported in the present experiments. While it was typically the case that the percentages of axon terminals in superficial layers exceeded that in deep layers (Ghashgaei et al. 2007), as seen in the present study, they reported a few areas, e.g., areas 11 and 12m, where the deep counts exceeded the superficial terminal counts. One apparent discrepancy between studies is the report of amygdala inputs that included layer IV in ventral parts of area 24 and parts of area 14c (referred to as area 025 by Ghashgaei et al. 2007). This discrepancy is somewhat misleading as both areas largely lack a layer IV (Carmichael and Price 1994), whereas there are some amygdala projections that appear to cross all layers of area 14c in a broad, columnar fashion (see Fig. 11*A*).

A number of frontal areas receive inputs from both the hippocampus and the amygdala. These sites included areas 13b, 13m, 14, 24a, 25, and 32. In almost all of these sites, however, there was limited, direct overlap as the respective projections terminated in different lamina. One example is area 13b (Fig. 11*B*, Table 1), which received the most consistent hippocampal inputs within the orbital region. Not only were the amygdala inputs to area 13b more restricted and lighter than the amygdala inputs to the other orbital areas, but the amygdala and hippocampal terminations in area 13b were in separate layers. Another example is area 32, where the hippocampal inputs appeared localized, whereas the dense amygdala projections reached the entire area. A more problematic site is area 25 as it contained many labeled fibers, especially at its caudal levels. However, by its mid-anterior–posterior levels, the fibers of passage in area 25 had largely disappeared and it could be seen that the amygdala terminations were concentrated in layer I whereas the hippocampal projections terminated in the remaining layers (Table 2). These examples highlight the complementary nature of the hippocampal and amygdala inputs (Fig. 14, Tables 1,2).

While the orbital cortex receives inputs from both the amygdala and hippocampus, these projections preferentially target different orbital sites. These differences may partly sustain the contrasting contributions of the macaque amygdala and hippocampus to fear expression (Chudasama et al 2009). It is only within the medial prefrontal cortex that projections from both structures consistently reach the same sites (Fig. 14). Consequently, these connection patterns reinforce the notion of distinct medial and orbital prefrontal networks (Price 1999; Kondo et al. 2005; Saleem et al. 2008). The precise degree of convergence within the medial prefrontal cortex remains uncertain as the respective projections are on different lamina, which may or may not involve terminations on the same dendrites. The amygdala terminations typically match the “descending” (superficial and deep layers) patterns of cortico-cortical connections described in sensory systems (Felleman and Van Essen 1991; see also Rockland and Pandya 1979). A few areas also contained the “lateral” (columnar) pattern of innervation (Felleman and Van Essen 1991). The frequent amygdala projections to layer II (see also Amaral and Price 1984; Ghashgaei et al. 2007) often overlapped with calbindin-positive inhibitory neurons in prefrontal cortex (Ghashgaei et al. 2007). These dense superficial inputs have been tentatively linked to roles in focusing attention on motivationally relevant stimuli (Ghashgaei et al. 2007). This dominant laminar pattern matches that seen in the amygdala projections back to sensory association cortices (Amaral and Price 1984), connections that are thought to have roles in emotional attention (Vuilleumier 2005). In contrast, the hippocampal projections typically match the “ascending” patterns of termination (Felleman and Van Essen 1991; see also Rockland and Pandya 1979). Finally, these contrasting termination patterns may also link to evidence concerning changes in the direction of signal transfer across lamina that distinguish sensory from mnemonic processing (Takeuchi et al. 2011).

There is considerable interest in how inputs from the amygdala and hippocampus to prefrontal cortex may be jointly involved in cognitive functions (e.g., Simons and Spiers 2003; Preston and Eichenbaum 2013; Rhodes and Murray 2013; Ruff and Fehr 2014). These medial temporal inputs fit with the general notion that prefrontal cortex has an integrative role requiring access to diverse information about both internal and external states (Miller and Cohen 2001). Particular examples include the ways in which emotional status and event information are integrated to give richer autobiographical memories (Fink et al. 1996; Talarico et al. 2004), alongside mechanisms that enable emotions to bias memories and affect consolidation processes (LaBar and Cabeza 2006). Unsurprisingly, these same temporal lobe connections with orbital and medial prefrontal cortex have also been linked to numerous dysfunctions, including obsessive–compulsive disorder (Milad and Rauch 2012), anxiety disorders (Bishop 2007), post-traumatic stress disorder (PTSD) (Phelps 2006), autism (Bachevalier and Loveland 2006), and schizophrenia (Delamillieure et al. 2002; Van Elset et al. 2005), all examples where connections involving both the hippocampus and amygdala are thought to contribute to the disorder. To take the example of PTSD, the interplay between hippocampal and amygdala connection with medial and orbital prefrontal cortex is centrally embedded in neural models of this disorder (Phelps 2006; Liberzon and Sripada 2007; Shin et al. 2007; Admon et al. 2013). The current finding of an area and lamina mismatch between the input sites from the amygdala and hippocampus is, therefore, relevant as it supports and informs those models that seek to distinguish hippocampal and amygdala interactions within prefrontal cortex in the predisposition and maintenance of PTSD (Admon et al. 2013). The present study also highlights the few areas of convergence between amygdala and hippocampal inputs, along with the significance of potential indirect routes from the hippocampus to the prefrontal cortex, which may include the many cortico-cortico connections between different prefrontal areas (Price 1999).

## Funding

The research was partly supported by a joint Royal Society/Wolfson Research Merit Award (MA09R2/HLL). Funding to pay the Open Access publication charges for this article was provided by a Royal Society Wolfson Research Merit Award to JPA.
